# Establishment of a new approach for quality evaluation of Pseudobulbus Cremastrae seu Pleiones (Shancigu) based on multicomponent analysis and anti-liver cancer pharmacological effects

**DOI:** 10.3389/fphar.2025.1544982

**Published:** 2025-06-12

**Authors:** Yuxin Cao, Zhuangzhuang Hao, Mengmeng Liu, Jingwen Xue, Yuqing Wang, Tingyue Jiang, Ge Zhang, Wenxin Fan, ChunGuo Wang, Jinli Shi

**Affiliations:** ^1^ School of Chinese Medica Materia, Beijing University of Chinese Medicine, Beijing, China; ^2^ Institute of Chinese Materia Medica, Beijing University of Chinese Medicine, Beijing, China

**Keywords:** Pseudobulbus Cremastrae seu Pleiones, different commercial specifications, spectrum–effect relationship, serum pharmacochemistry, anti-liver cancer effective component combination, quality evaluation

## Abstract

**Background:**

Pseudobulbus Cremastrae seu Pleiones (Shancigu), a traditional Chinese medicine (TCM), has been extensively used in clinical practice for the treatment of various tumors, particularly liver cancer. Shancigu is classified into two commercial specifications—“Maocigu” and “Bingqiuzi”—which exhibit significant differences in appearance, chemical composition, and price, posing challenges for the quality control of medicinal materials.

**Purpose:**

The aim of this study was to clarify the quality evaluation indicators based on the anti-liver cancer active components in Shancigu and to establish a reliable quality evaluation method to preliminarily assess the quality of Shancigu from different commercial specifications and production areas.

**Methods:**

Twenty-six batches of Shancigu samples were collected. High-performance liquid chromatography (HPLC) was used to establish fingerprint spectra. *In vitro* anti-liver cancer pharmacological effect indicators were analyzed using the CCK-8 assay and scratch wound healing assays. Through spectrum–effect relationship analysis, serum pharmacochemistry analysis, and *in vitro*/*in vivo* anti-liver cancer activity evaluation, the effective component combinations of Bingqiuzi and Maocigu were identified and validated. Gray relational analysis (GRA) and the technique for order preference by similarity to ideal solution (TOPSIS) were subsequently applied to assess the quality of Shancigu based on their different specifications and origins.

**Results:**

Eleven key anti-liver cancer active components from Shancigu were screened and confirmed, namely, malic acid, citric acid, 2-isobutylmalic acid, gastrodin, batatasin III, 2-*p*-hydroxybenzyl-5,3′-dihydroxy-3-methoxybibenzyl, coelonin, 1-*p*-hydroxybenzyl-2,7-dihydroxy-4-methoxyphenanthrene, blestriarene A, blestriarene B, and monbarbatain A. These components are present in Bingqiuzi and Maocigu in different proportions, and the anti-liver cancer pharmacological effects of the effective component combinations were found to be equivalent to those of the original materials, both *in vitro* and *in vivo*. These 11 components can be used as indicators for evaluating the quality of Shancigu. Quality evaluations revealed no significant differences between Bingqiuzi and Maocigu. For Bingqiuzi, medicinal materials produced in Guizhou and Yunnan were of better quality; for Maocigu, those from Guizhou and Sichuan were superior.

**Conclusion:**

In this study, we established quality evaluation criteria for Shancigu and developed an innovative method to comprehensively assess the quality of Shancigu from different commercial specifications and production regions. By integrating component analysis with anti-liver cancer activity assessment, this research provides a valuable reference for the quality evaluation of other Chinese medicinal materials.

## 1 Introduction

Pseudobulbus Cremastrae seu Pleiones (Shancigu) is derived from the dried pseudobulbs of the orchid species *Cremastra appendiculata* (D.Don) Makino, *Pleione bulbocodioides* (Franch.) Rolfe, and *Pleione yunnanensis* Rolfe (Chinese Pharmacopoeia Commission, 2020). In both ancient and modern medical literature studies, Shancigu is recorded as entering the liver and spleen meridians and is characterized by its therapeutic effects, such as clearing heat, detoxifying, resolving phlegm, and dissipating nodules. It has been acclaimed as a “sacred herb for resolving phlegm” (Chen, 1983) and is primarily used in the treatment of malignant tumors, including liver cancer, lung cancer, and breast cancer (Chen et al., 2023). Shancigu was first documented in the *Tang Dynasty’s Supplement to Materia Medica*, where it was noted for treating “carbuncles, sores, fistulas, scrofula, and tuberculosis.” In the *Compendium of Materia Medica*, it is further described as treating “abscesses, ulcers, fistulas, and scrofula.” Since 1998, its use in the treatment of liver cancer has been increasingly reported (Zhang, 1998). According to the theory of traditional Chinese medicine (TCM), liver cancer falls under the categories of “Ji Ju” (accumulations) and “Zheng Jia” (masses), with its pathogenesis primarily attributed to the obstruction of the liver’s ascending function. The stagnation of phlegm, blood stasis, and toxic heat in the liver leads to the formation of palpable masses. Therefore, clinical treatment often emphasizes clearing heat and toxins, resolving phlegm, and dispersing blood stasis (Peng et al., 2022).

Shancigu mainly contains organic acids, glycosides, phenanthrenes, and bibenzyl components (Liu et al., 2021), which exhibit various pharmacological activities, including antitumor, antibacterial, and antioxidant effects (Jiang et al., 2022). Militarine, the most abundant glycoside in Shancigu, is currently used as a quality marker (Yang et al., 2020); however, its specific contribution to medicinal efficacy remains unclear. Although phenanthrenes demonstrate potent antitumor activity *in vitro* (Dong et al., 2013; Wang et al., 2013), their extremely low content renders them unsuitable as independent quality indicators. In addition, organic acids and bibenzyls are also important components of Shancigu, but their pharmacological roles have not been fully elucidated. Currently, the material basis underlying the anti-liver cancer pharmacological effects of Shancigu remains to be clarified, and it is unscientific to control its quality based solely on the content of a single or a few components. Therefore, it is crucial to establish a scientific and effective quality evaluation approach based on the correlation of “efficacy-multicomponents.”

The *General Rules for Commercial Specifications and Grades of Chinese Medicinal Materials* (SB/T 11,173-2016) stipulate that commercial specifications serve as the basis for distinguishing different trading categories of Chinese medicinal materials during circulation, encompassing criteria such as botanical origin, production area, and medicinal material characteristics. Shancigu is divided into two commercial specifications in the market: “Maocigu” (including CA, also named *Cremastrae Pseudobulbus*) and “Bingqiuzi” (including PB and PY, also named *Pleiones Pseudobulbus*), according to botanical origin, with significant differences in price, appearance, and chemical composition (Hao et al., 2023). Thus, the quality of Shancigu is influenced not only by the production area but also by its commercial specifications. However, the current *Chinese Pharmacopoeia* (2020 edition) lacks critical quality evaluation indicators to distinguish between these specifications, and existing studies have not addressed this differentiation.

Effective component combinations in TCM are key factors that link efficacy and quality, reflecting the holistic therapeutic effect of TCM. Currently, the majority of studies evaluate TCM quality by measuring the content of one or multiple chemical components along with specific *in vitro* efficacy indicators. However, this approach has significant limitations, primarily because it struggles to reveal the intrinsic relationship between chemical components and therapeutic effects. To address this issue, it is necessary to select appropriate efficacy indicators based on disease syndrome characteristics, integrate chemical components with therapeutic outcomes, and subsequently identify the effective component combinations that genuinely reflect drug efficacy. These combinations should then serve as the core criteria for TCM quality evaluation. This approach demonstrates that there is an inherent and organic connection among components, therapeutic effects, and disease syndromes, with the content of effective component combinations determining the construction of the quality evaluation system. Therefore, it is crucial to clarify the effective component combination for anti-liver cancer in Shancigu for evaluating its quality.

As a quality evaluation indicator, multicomponents related to efficacy can more objectively reflect the overall quality and the synergistic characteristics of multiple components in TCM. Extensive studies on the spectrum–effect relationship have been conducted to elucidate the pharmacodynamic substance components of TCM while preserving its holistic and multicomponent synergistic nature (Liu et al., 2024). The drug-containing serum after oral administration of TCM is a critical focus of research, as the serum-absorbed components are considered to represent the final active components (Deng et al., 2024). By integrating spectrum–effect relationship analysis with serum pharmacochemistry, the selected effective component combinations can serve as quality evaluation indicators that comprehensively reflect the intrinsic quality of TCM. On this basis, Gray relational analysis (GRA) can be used to evaluate the contribution of each component to drug efficacy (Li et al., 2024), whereas the technique for order preference by similarity to ideal solution (TOPSIS) can assign weights to multiple indicators and enable the comprehensive quality evaluation of multiple batches of medicinal materials (Zhang et al., 2023). Combining these two methods allows for weighting the efficacy contribution of the indicators in a multi-indicator evaluation, thereby rendering the quality evaluation model more scientific and rational.

Therefore, in this study, we developed an integrated pharmacological approach by combining spectrum–effect relationship analysis, serum pharmacochemistry, and efficacy validation guided by anti-liver cancer activity, aiming to elucidate the effective component combinations and clarify the quality evaluation indicators of Shancigu with different commercial specifications. Furthermore, in this study, we established a multi-index quality evaluation method for TCM under efficacy empowerment by integrating the GRA and TOPSIS methods and evaluated the quality of 26 batches of Shancigu from different commercial specifications and production areas.

## 2 Materials and methods

### 2.1 Materials and reagents

The chromatographic/MS-grade methanol, acetonitrile, and formic acid utilized in this research were all purchased from Fisher Chemical (Waltham, MA, United States). The standard substances, including gastrodin, dactylorhin A, militarine, batatasin III, 2-(*p*-hydroxybenzyl)-5,3’-dihydroxy-3-methoxybenzyl, blestriarene A, blestriarene B, monbarbatain A, 1-hydroxybenzyl-2,7-dihydroxy-4-methoxyphenyl, coelonin, malic acid, and citric acid, were all purchased from Chengdu Mansite Biotech Co., Ltd. (Chengdu, China). Loroglosin was purchased from Chengdu Purechem-standard Co., Ltd. (Chengdu, China). The purity of each reference component was over 98%, as analyzed by HPLC.

Fetal bovine serum (FBS) was purchased from Corning Co., LTD. (New York, United States). Dulbecco’s modified Eagle medium (DMEM) and antibiotics (penicillin and streptomycin) were all purchased from Gibco Co., Ltd. (Gibco-BRL, Grand Island, NY, United States). Dimethyl sulfoxide (DMSO) was obtained from Solarbio Technology Co., Ltd. (Beijing, China). Sorafenib was obtained from Shanghai Yuanye Biotechnology Co., Ltd. (Shanghai, China). A CCK-8 kit was obtained from New Cell & Molecular Biotech Co., Ltd. (Suzhou, China).

Human hepatocellular carcinoma cells HepG2 and Huh7, and the mouse hepatocellular carcinoma cell H22 lines were all purchased from BNCC Technology Co., Ltd. (Beijing, China).

### 2.2 Collection of samples and preparation of the Shancigu extract

The details of the 26 batches of Shancigu samples are shown in Supplementary Table S1. All dried Shancigu samples were collected from different planting bases across four main provinces, namely, Guizhou, Yunnan, Sichuan, and Guangxi provinces. Among them, samples S1–S14 were Bingqiuzi, and samples S15–S26 were identified as Maocigu. They were identified as the dried pseudobulbs of *C. appendiculata* (D. Don) Makino, *P. bulbocodioides* (Franch.) Rolfe, and *P. yunnanensis* Rolfe by Professor Jinli Shi of the Beijing University of Chinese Medicine. All samples were stored in dark, dry, and cool places.

Each batch of Shancigu was ground into powder and passed through a 60-mesh sieve. The samples were then mixed with 70% ethanol at 20 times their volume, extracted by reflux for 90 min, and continuously extracted twice. All filtrates were combined, concentrated into a thick paste, and freeze-dried. The Shancigu extracts were obtained and stored in a refrigerator at 4°C. The extraction rates of the 26 batches of Shancigu extracts are shown in Supplementary Table S1.

### 2.3 Animals and establishment of H22 xenograft tumor models

Eight-week-old BALB/c mice (male, 18–22 g) were used in a tumor-burden experiment and were purchased from Beijing Vital River Laboratory Animal Technology Co., Ltd., with animal license number SCXK (Beijing) 2021-0006. For the experiment, the mice were maintained in a specific pathogen-free (SPF) environment at the Beijing University of Traditional Chinese Medicine, with access to standard rodent chow and water, under 12-h light–dark cycles at a constant room temperature. All animal procedures conformed to the requirements of international ethics for laboratory animals, and the ethics approval number was BUCM-2023032001-1045.

The experiment was performed after 5 days of adaptation. H22 cells suspended in PBS at a density of 1 × 10^7^ cells/mL were injected (0.1 mL) into the right axillary subcutaneous tissue of the BALB/c mice. The H22 xenograft tumor model was considered successfully established when the average tumor volume reached 100 mm^3^.

### 2.4 Study of the spectrum–effect relationship

#### 2.4.1 HPLC fingerprint analysis

The high-performance liquid chromatography (HPLC) system, LC-20A (Shimadzu Co., Ltd., Japan), was utilized for the chromatographic analysis. To acquire accurate chromatographic results, the following optimized chromatographic conditions were applied: separation was carried out on a Zorbax SB-C18 column (250 mm × 4.6 mm, 5 μm), using acetonitrile as mobile phase A and ultrapure water as mobile phase B, with a gradient elution program as follows: 0–4 min, 5% A; 4–5 min, 5%–20% A; 5–35 min, 20%–33% A; 35–70 min, 33%–52% A; 70–72 min, 52%–95% A; and 72–80 min, 95% A, at a flow rate of 1 mL/min. The injection volume was 10 μL, and the detection wavelength was set at 200 nm. The column temperature was maintained at 30°C.

The chromatographic data from 26 batches of Shancigu samples were input into the Similarity Evaluation System for TCM Chromatographic Fingerprints (2012 edition), and similarity tests were conducted separately on the 14 batches of Bingqiuzi and the 12 batches of Maocigu.

#### 2.4.2 Cell culture

HepG2 and Huh7 cells were cultured in DMEM supplemented with 10% FBS and antibiotics (100 U/mL penicillin and 100 μg/mL streptomycin) at 37°C in a humidified atmosphere with 5% CO_2_.

#### 2.4.3 Cell viability assay

The CCK-8 assay was carried out to determine cell viability. Cells were seeded into 96-well plates (6 × 10^3^ cells per well), with each experiment repeated six times. After incubation at 37°C for 24 h, the cells were treated with various concentrations of Shancigu for 24 or 48 h. Sorafenib was used as a positive control. Next, a 10% CCK-8 solution was added to each well and incubated at 37°C for 1 h. The absorbance of each well was then measured using the BioTek Epoch2 microplate reader [Corent (Beijing) Technology Co., Ltd., China] at 450 nm, and the inhibition rate was calculated using the following formula: Inhibition (%) = [(A_treatment_ - A_blank_)/(A_control_ - A_blank_)] ×100%.

#### 2.4.4 Wound healing assay

Cells were cultured in 96-well plates and grown to 80%–90% confluence. The cells were then scratched using an automatic scratch tool, followed by treatment with the appropriate samples containing low-serum culture medium (2% FBS) for 48 h. The scratches were photographed using the IncuCyte S3 live cell dynamic imaging system at 0, 24, 36, and 48 h. The scratch width was then statistically analyzed.

#### 2.4.5 Spectrum–effect relationship analysis

GRA is a method used to assess the extent of similarity or difference between the factors of two systems over time or across different objects (Du et al., 2021). Bivariate correlation analysis (BCA) can predict and explain the linear relationship between two variables (Qiao et al., 2021). The inhibition rates of cancer cell viability and migration ability were chosen as the reference series, and common peak areas accounting for more than 0.02% of the total peak area were selected as the comparative series. GRA and BCA were performed using SPSS 21.0 to evaluate the relationship between the fingerprint and pharmacological effects. Based on the results of GRA and BCA, the correlation between the pharmacodynamic indexes and the chromatographic peaks was analyzed, and the active constituents were screened.

### 2.5 Study of the serum pharmacochemistry

#### 2.5.1 Collection of serum samples

Except for the control group, mice with tumors were randomly assigned to the model group, Bingqiuzi treatment group (samples collected after administration at 0.5 h, 1 h, 2 h, and 4 h), and Maocigu treatment group (samples collected after administration at 0.5 h, 1 h, 2 h, and 4 h) (all samples were measured based on the amount of crude medicinal botanical drugs for human use: 12 g/ 60 kg^-1^), with five mice in each group at each time point. Blood was collected from the eyeballs of the mice and centrifuged at 3,000 rpm at 4°C for 15 min. The serum from the four time points after administration was equally mixed and stored at −80°C until analysis.

#### 2.5.2 Preparation of mouse serum samples

A 500-μL serum sample was placed in an Eppendorf (EP) tube and then mixed with 1000 μL of acetonitrile and 1000 μL of methanol by vortex mixing. The mixture was centrifuged at 12,000 r/min for 15 min, and the supernatant was collected and dried under nitrogen gas. Then, 100 μL of 60% acetonitrile was added to dissolve the residue. The solution was centrifuged again at 12,000 r/min for 15 min, and the supernatant was collected for analysis.

#### 2.5.3 UHPLC-Q-Exactive Orbitrap MS method for serum pharmacochemistry analysis

The serum samples were analyzed using a Thermo UHPLC system connected to a Thermo Q-Exactive-Orbitrap-MS system (Thermo Fisher Scientific, Waltham, MA, United States). Chromatographic separation was carried out using a Waters ACQUITY UPLC HSS T3 column (2.1 mm × 100 mm, 1.8 μm). The mobile phase consisted of deionized water with 0.1% formic acid (A) and acetonitrile (B). The gradient elution procedure was as follows: 0–4 min, 2% B; 4–5 min, 2%–5% B; 5–20 min, 5%–16% B; 20–50 min, 16%–45% B; and 50–60 min, 45%–80% B. The column temperature was maintained at 30°C. The flow rate was set at 0.3 mL/min, and the injection volume was 2 μL. Mass spectrometry was performed in the negative ion mode over a mass range of m/z 120–1500. The ESI source parameters were as follows: source temperature, 400°C; ion spray voltage, 3.5 kV; and capillary temperature, 320°C. Both the sheath gas and the auxiliary gas were nitrogen and were maintained at 40 arb and 10 arb, respectively. The collision energy gradient was set at 20, 40, and 60 eV. The resolution of the first level was 70,000, and that of the second level was 17,500. All operations mentioned above were performed using Xcalibur software.

### 2.6 Pharmacological effect validation *in vitro*

The pharmacological effects of the active and effective component combinations were validated in liver cancer cells. A series of different concentration media containing the medicines were added to HepG2 and Huh7 cells and treated for 48 h (all samples we administered were adjusted to a pH of 7.2–7.4 using Na_2_CO_3_ and NaOH). Cell viability (%) and cell migration rates (%) were calculated.

### 2.7 Pharmacological effect validation *in vivo*

Except for the control group, the mice bearing H22 xenograft tumors were randomly assigned to six groups (10 mice/group), namely, the model group, the positive drug (sorafenib: 30 mg/kg/d, i.g.) group, the MCG-9 (275.5 mg/kg/d, i.g.) group, the MCGC (78.4 mg/kg/d, i.g.) group, the BQZ-3 (337.3 mg/kg/d, i.g.) group, and the BQZC (130.5 mg/kg/d, i.g.) group (all measured based on the amount of crude medicinal herbs for human use: 12 g/ 60 kg^-1^). The mice were treated via intragastric administration for 10 days, once daily. The body weights of the mice and the volumes of the tumors were measured every 3 days. Finally, the mice were sacrificed by cervical dislocation, and the tumors were collected for further analysis.

### 2.8 Determination of active component content in effective component combination

The lyophilized powders of 26 batches of Shancigu were dissolved in 60% acetonitrile to obtain a solution with a concentration of 160 mg mL^-1^ (calculated based on the amount of crude medicinal herbs).

Seven standards were weighed accurately using an analytical balance: monbarbatain A, 166.70 µg; blestriarene A, 166.70 µg; blestriarene B, 166.70 µg; coelonin, 200.00 µg; 2,7-dihydroxy-1-(4-hydroxybenzyl)-4-methoxyphenanthrene, 200.00 µg; 2-(*p*-hydroxybenzyl)-3′,5-dihydroxy-3-methoxybibenzyl, 50 μg; and batatasin III, 11 µg. The standards were dissolved completely in a 10-mL volumetric flask with 60% acetonitrile to prepare a stock solution, which was then diluted in a gradient to produce seven standard samples. These samples were analyzed using the abovementioned HPLC method, and a standard curve was plotted.

Additionally, four standards were weighed accurately: gastrodin, 5 mg; 2-isobutylmalic acid, 1.25 mg; malic acid, 50 mg; and citric acid, 5 mg. Gradient dilution was performed as described above. These samples were analyzed using the above mentioned UHPLC-MS method, and a standard curve was plotted.

### 2.9 Establishment of a Shancigu comprehensive quality evaluation model based on GRA and TOPSIS

The comprehensive anti-liver cancer pharmacological effect indicators were calculated by weighting the standardized data of the four *in vitro* anti-liver cancer pharmacological effect indicators. GRA was performed to calculate the contribution of each component to the pharmacological effects based on the content of 11 components in 20 batches of Shancigu and the comprehensive pharmacological effect index.

Based on the standardized content data of the 11 active components in the 26 batches of Shancigu as the indicators, the contribution of each component to the comprehensive pharmacological effects against liver cancer was used as the weight coefficient, and the quality of the 26 batches of Shancigu was evaluated using TOPSIS analysis.

### 2.10 Statistical analysis

Data analysis was performed using SPSS 21.0 software. All data are expressed as mean ± SD (standard deviation). One-way ANOVA (and nonparametric analysis) was used for comparisons between groups. A *p*-value of <0.05 was considered statistically significant. Bar graphs were created using GraphPad Prism 5 software to visualize the pharmacological and chemical data.

## 3 Results

### 3.1 Fingerprint acquisition and identification of component peaks

The method validation results showed that the RSD (%) of the relative peak area (RPA) for instrument precision, method repeatability, and method stability over 24 h were all below 2.00%, and the RSD (%) of the relative retention time (RRT) for these parameters was all below 1.00%. This indicates that the method was accurate and reliable, and could be applied to establish an HPLC fingerprint for sample analysis.

**FIGURE 1:**
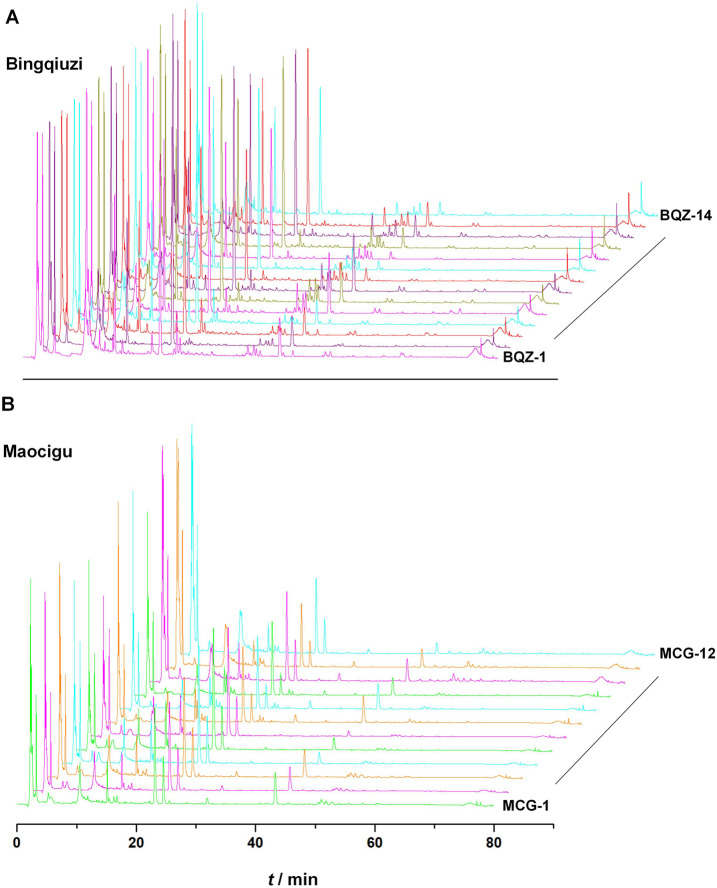
HPLC fingerprints of 14 batches of Bingqiuzi samples **(A)** and 12 batches of Maocigu samples **(B)**.

Fingerprint spectra for different commercial specifications of Shancigu were established. Among them, 14 batches of Bingqiuzi were matched with 46 common peaks, with similarity ranging from 0.986 to 0.997; 12 batches of Maocigu were matched with 42 common peaks, with similarity ranging from 0.969 to 0.996 (Figure 1) (Supplementary Table S2). The peak areas of the common peaks are shown in Supplementary Tables S3, S4. Among them, Maocigu has only one botanical origin, *C. appendiculata* (D.Don) Makino, indicating good chemical consistency within this origin. Another commercial specification, Bingqiuzi, includes two botanical origins: *P. bulbocodioides* (Franch.) Rolfe and *P. yunnanensis* Rolfe. Nevertheless, its chemical consistency remains relatively high, likely because both species belong to the same genus (*Pleione*), suggesting only minor differences in chemical composition within this genus.

**FIGURE 2:**
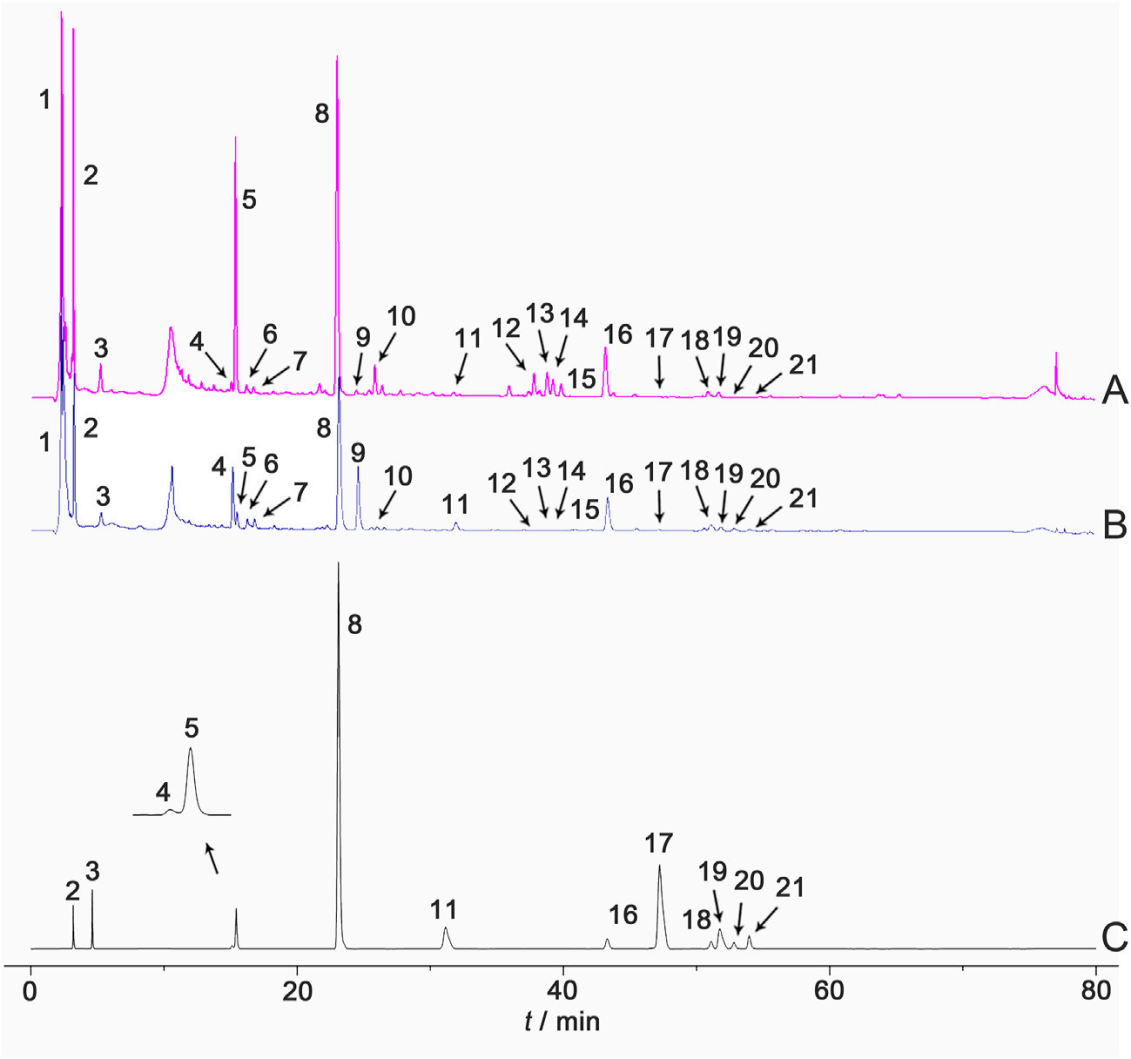
HPLC reference fingerprints of **(A)** Bingqiuzi, **(B)** Maocigu, and **(C)** mixed control samples. Note: (1) malic acid, (2) gastrodin, (3) 2-isobutylmalic acid, (4) loroglossin, (5) dactylorhin A, (6) gymnoside, (7) gymnoside II, (8) militarine, (9) 1,4-di (4-*β*-D-glucopyranosyloxybenzyl)-2-benzylmalate diester, (10) shancigusin H, (11) coelonin, (12) gymnoside V, (13) gymnoside VI, (14) gymnoside IV, (15) gymnoside V isomer, (16) batatasin III, (17) 2,7-dihydroxy-1-(4-hydroxybenzyl)-4-methoxyphenanthrene, (18) blestriarene A, (19) 2-(*p*-hydroxybenzyl)-3’,5-dihydroxy-3-methoxybibenzyl, (20) blestriarene B, and (21) monbarbatain **(A)**.

These results indicate that the chemical characteristics of Shancigu samples with the same commercial specification were highly similar. Through the identification of chemical components by LC-MS during the previous period, combined with the comparison against reference materials and literature data, a total of 21 component peaks were identified, including gastrodin, batatasin III, and monbarbatain A (Figure 2).

### 3.2 Detection of anti-liver cancer pharmacological effect indicators *in vitro*

One of the important characteristics of liver cancer cells is the acceleration of cell metabolism to meet the energy demand of rapid proliferation. The measurement of cell proliferation activity can better evaluate the effect of drug treatment on the proliferation of liver cancer cells. Moreover, liver cancer is highly invasive and metastatic, and the cell migration ability is an important indicator of invasion and metastasis of liver cancer cells *in vivo*.

**FIGURE 3:**
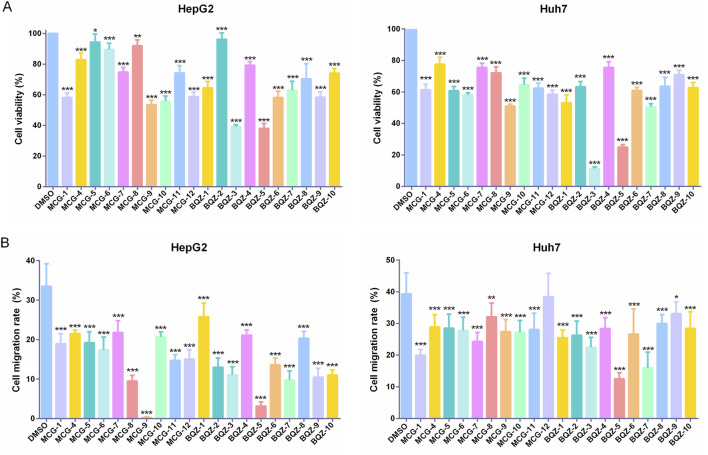
Effect of 10 batches of Bingqiuzi samples and 10 batches of Maocigu samples on anti-liver cancer activity *in vitro*. **(A)** Effect on the proliferation activity of HepG2 and Huh7 cells. **(B)** Effect on the migration ability of HepG2 and Huh7 cells. Data are presented as mean ± SD, *n* = 6; ^*^ *P* < 0.05, ^**^ *P* < 0.01, and ^***^ *P* < 0.001, treatment group vs control.

Therefore, the effects of Shancigu on the proliferation and migration abilities of HepG2 and Huh7 cells were detected as *in vitro* evaluation indexes for anti-liver cancer pharmacological effects to discover candidate active components. As depicted in Supplementary Figure S1, MCG-1 from Shancigu samples decreased the viability of HepG2 and Huh7 cells in a time- and concentration-dependent manner at concentrations ranging from 0.125 to 3 mg/mL, with IC_50_ values of 1.208 mg/mL and 0.957 mg/mL at 48 h, respectively. A concentration of 1 mg/mL of MCG-1 showed significant inhibition of the two liver cancer cell lines and was chosen for the cytotoxicity evaluation of 20 batches of Shancigu. Subsequently, a drug concentration with low cytotoxicity (0.5 mg/mL) was selected to measure cell migration activity. The chemical variation among different batches of Shancigu samples evinced diverse inhibitory effects on cell proliferation and migration activity (Figures 3A,B). The results of the *in vitro* pharmacodynamic indexes for the 20 batches of Shancigu are shown in Supplementary Table S5. The results demonstrated that the different batches exhibited varying anti-liver cancer activities. The anti-liver cancer activity of Bingqiuzi was slightly better than that of Maocigu; however, no significant difference was observed. Among them, the BQZ-3, BQZ-5, BQZ-7, and MCG-9 batches showed greater abilities to inhibit cell proliferation and cell migration.

### 3.3 Spectrum–effect relationship analysis

Gray correlation degrees and correlation coefficients between four pharmacological indicators and each common peak of Shancigu in different commercial specifications were calculated using GRA and BCA to determine the contribution of each component peak to the pharmacodynamic indexes and to screen out the anti-liver cancer effective components in different commercial specifications of Shancigu.

#### 3.3.1 Gray relational analysis

The greater the gray correlation degree of a component peak, the stronger its correlation with the drug effect; however, it is not possible to determine whether the correlation is positive or negative. As shown in Supplementary Table S6, the gray correlation degrees between the common peaks of Bingqiuzi and the inhibition rates of HepG2 and Huh7 cell viability and migration ranged from 0.612 to 0.818, 0.612 to 0.821, 0.608 to 0.820, and 0.600 to 0.773, respectively. Among these, the top-ranked components were dactylorhin A, 1-*p*-hydroxybenzyl-2,7-dihydroxy-4-methoxyphenanthrene, blestriarene A, blestriarene B, and monbarbatain A. As shown in Supplementary Table S7, the gray correlation degrees between the common peaks of Maocigu and the inhibition rates of HepG2 and Huh7 cell viability and migration ranged from 0.633 to 0.883, 0.712 to 0.863, 0.686 to 0.849, and 0.664 to 0.857, respectively. Among these, the top-ranked components were loroglossin, 2-isobutylmalic acid, gastrodin, gymnoside, coelonin, 2-*p*-hydroxybenzyl-5,3′-dihydroxy-3-methoxybibenzyl, and blestriarene A.

#### 3.3.2 Bivariate correlation analysis

The greater the absolute value of a component peak’s correlation coefficient, the stronger its correlation with the drug effect. A positive coefficient indicates a positive correlation, whereas a negative value indicates a negative correlation. As shown in Supplementary Table S8, there were 16 peaks with positive correlation coefficients with the common peaks of Bingqiuzi and the HepG2 cell viability inhibition rate, including b4 (loroglossin), b29, b33, b34 (1-*p*-hydroxybenzyl-2,7-dihydroxy-4-methoxyphenanthrene), and b39 (monbarbatain A). There were 11 peaks with positive correlation coefficients with the Huh7 cell viability inhibition rate, including b32, b27 (gymnoside V isomer), b3, b19, and b39 (monbarbatain A). There were 10 peaks with positive correlation coefficients with the migration inhibition rate of HepG2 cells, including b25 (gymnoside VI), b24, b5 (dactylorhin A), b35, and b30. There were eight peaks with positive correlation coefficients with the migration inhibition rate of Huh7 cells, including b27 (gymnoside V isomer), b19, b18, b32, and b38 (blestriarene B).

As shown in Supplementary Table S9, there were 31 peaks with positive correlation coefficients with the common peaks of Maocigu and the HepG2 cell viability inhibition rate, including m2 (2-isobutylmalic acid), m37, m33 (blestriarene A), m1 (gastrodin), and m15 (1,4-di (4-*β*-D-glucopyranosyloxybenzyl)-2-benzylmalate diester). There were 28 peaks with positive correlation coefficients with the Huh7 cell viability inhibition rate, including m36 (monbarbatain A), m37, m33 (blestriarene A), m8 (gymnoside Ⅱ), and m35 (blestriarene B). There were 27 peaks with positive correlation coefficients with the migration inhibition rate of HepG2 cells, including m3, m5 (loroglossin), m4, m9, and m13. There were 13 peaks with positive correlation coefficients with the migration inhibition rate of Huh7 cells, including m37, m1 (gastrodin), m30 (1-*p*-hydroxybenzyl-2,7-dihydroxy-4-methoxyphenanthrene), m36 (monbarbatain A), and m27.

By combining GRA and BCA, we removed the peaks with negative correlation coefficients that could adversely affect the pharmacological effect indicators and took the intersection of the top 10 absolute values of correlation coefficients and gray correlation coefficients among the remaining peaks to screen active components (Table 1). The results showed that there was a higher correlation between the pharmacological indicators and the phenanthrenes and glycosides, such as monbarbatain A and gymnoside V isomer in Bingqiuzi; the correlation of glycosides such as gastrodin, phenanthrenes such as blestriarene A, along with 2-hydroxybenzyl-5,3′-dihydroxy-3-methoxybibenzyl and 2-isobutylmalic acid was higher in Maocigu.

**TABLE 1:** Active component screening based on GRA and BCA.

GRA (top ten) ∩ BCA (top ten)	Screened active components (peaks)			
	HepG2 proliferation inhibition	HepG2 migration inhibition	Huh7 proliferation inhibition	Huh7 migration inhibition
Bingqiuzi	b4, b33, b34, b39, b43, and b15	b5, b25, b24, b6, b32, and b22	b3, b19, b38, b4, b27, b33, and b39	b27, b32, b39, b19, b38, and b3
	b3, b4, b5, b6, b15, b19, b22, b24, b25,b27, b32, b33, b34, b38, b39, and b43			
Maocigu	m32, m3, m8, m36, m35, and m29	m33, m8, and m34	m5, m3, m34, m14, m35, m15, m23, and m36	m1, m30, m36, m37, m27, m23, m2, m29, m33, and m39
	m1, m2, m3, m5, m8, m14, m15, m23, m27, m29, m30, m32, m33, m34, m35, m36, m37, and m39			

### 3.4 Serum pharmacochemistry analysis

The results were analyzed using Xcalibur 4.0 software. TIC chromatograms of blank serum, model serum, and drug serum under negative ion mode are shown in Supplementary Figure S2. Using primary and secondary mass spectrometry data, a total of 33 compounds were identified through database (PubChem, m/z Cloud, ChemSpider, MassBank, *etc*.) and literature comparison (Table 2), including 7 glycosides, 16 organic acids, and 10 phenanthrenes and bibenzyls. The abundance of glycosides was relatively low, whereas that of gastrodin and organic acids was high. This may be due to the poor stability of glycosides in Shancigu, which are connected by ester bonds between gastrodin and organic acids, such as 2-isobutylmalic acid, resulting in the destruction of ester bonds upon entry into the bloodstream.

**TABLE 2:** Characterization of components in mouse serum after oral administration based on UPLC-Q-Exactive Orbitrap-MS.

NO.	RT/min	Adduct	Theoretical m/z (Da)	Mass error (ppm)	Characteristic ion	Component formula	Component	Source	Type
1	1.09	[M-H]^-^	191.01971	−0.05	111.01、87.01、85.03、191.02、133.05	C_6_H_8_O_7_	Isocitric acid	B/M	Organic acid
2	1.23	[M-H]^-^	133.01418	−0.45	115.00、71.01、133.01	C_4_H_6_O_5_	Malic acid	B/M	Organic acid
3	1.33	[M-H]^-^	147.02995	0.41	147.03、129.02、115.00、87.01、85.03	C_5_H_8_O_5_	2-Methylmalic acid	B/M	Organic acid
4	1.42	[M-H]^-^	117.01929	−0.34	73.03、117.02	C_4_H_6_O_4_	Methylmalonic acid	B/M	Organic acid
5	1.56	[M-H]^-^	161.04549	−0.31	57.03、99.05、73.03、73.03、131.03	C_6_H_10_O_5_	2-Ethylmalic acid	B/M	Organic acid
6	1.64	[M-H]^-^	191.01967	−0.26	111.01、87.01、85.03、191.02、133.05	C_6_H_8_O_7_	Citric acid	B/M	Organic acid
7	3.27	[M-H]^-^	137.02428	−0.95	108.02、93.03、80.03	C_7_H_6_O_3_	*o*-Hydroxybenzoic acid	B	Organic acid
8	4.11	[M + HCOO]^-^	331.10364	0.57	123.05、161.05、285.10、331.10	C_13_H_18_O_7_	Gastrodin	B/M	Glycoside
9	5.7	[M-H]^-^	137.02432	−0.66	108.02、93.03、80.03	C_7_H_6_O_3_	*m*-Hydroxybenzoic acid	B	Organic acid
10	7.11	[M-H]^-^	205.07161	−0.73	115.08、205.07、129.06、143.07、72.99	C_8_H_14_O_6_	2-Isobutyltartaric acid	B/M	Organic acid
11	9.66	[M-H]^-^	175.06114	−0.29	115.04、175.06、146.96、113.06、85.07	C_7_H_12_O_5_	2-Isopropylmalic acid	B/M	Organic acid
12	12.46	[M-H]^-^	239.05623	0.50	239.06、179.03、177.06、133.07	C_11_H_12_O_6_	Eucomic acid	B/M	Organic acid
13	12.95	[M-H]^-^	351.12976	0.26	127.08、171.07、351.13	C_14_H_24_O_10_	Dactylorhin C	B/M	Glycoside
14	13.12	[M-H]^-^	121.02953	0.25	121.03、108.02	C_7_H_6_O_2_	*p*-Hydroxybenzaldehyde	B/M	Organic acid
15	13.51	[M-H]^-^	189.07695	0.58	129.06、189.08、127.08、99.08	C_8_H_14_O_5_	2-Isobutylmalic acid	B/M	Organic acid
16	13.9	[M-H]^-^	619.22552	1.89	153.06、439.16、123.05	C_27_H_40_O_16_	Dactylorhin E	B	Glycoside
17	13.94	[M-H]^-^	473.16638	−0.13	115.08、159.07、143.07	C_21_H_30_O_12_	Coelovirin B	M	Glycoside
18	14.02	[M-H]^-^	163.04010	0.25	119.05、163.04	C_9_H_8_O_3_	*p*-Hydroxycinnamic acid	B/M	Organic acid
19	14.36	[M-H]^-^	223.06119	0.00	223.06、163.04、161.06、133.06	C_11_H_12_O_5_	2-Benzylmalic acid	B/M	Organic acid
20	14.48	[M + C_12_H_15_O_12_]^-^	833.23035	0.62	113.02、657.20、466.14、175.02、833.23	C_30_H_26_O_6_	Blestriarene A	B/M	Phenanthrene
21	14.54	[M + C_12_H_15_O_12_]^-^	699.19275	−0.43	523.16、113.02、253.09、175.02、347.13	C_22_H_20_O_4_	1-*p*-Hydroxybenzyl-4,7-dihydroxy-2-methoxy-9,10-dihydrophenanthrene	B/M	Phenanthrene
22	14.63	[M + C_12_H_15_O_12_]^-^	697.17792	0.75	521.15、113.02、175.02、251.07、345.11	C_22_H_18_O_4_	2,7-Dihydroxy-1-(4-hydroxybenzyl)-4-methoxyphenanthrene	B/M	Phenanthrene
23	14.63	[M + C_12_H_15_O_12_]^-^	831.21423	0.06	113.02、655.18、479.15、175.02	C_30_H_24_O_6_	Blestriarene B	B/M	Phenanthrene
24	14.72	[M-H]^-^	457.17194	0.90	123.05、129.06、127.08、153.06、285.10	C_21_H_30_O_11_	Gymnoside	B/M	Glycoside
25	14.74	[M + C_12_H_15_O_12_]^-^	829.19922	0.83	113.02、653.17、477.14、175.02	C_30_H_22_O_6_	Monbarbatain A	B/M	Phenanthrene
26	14.76	[M + C_12_H_15_O_12_]^-^	859.20831	−0.92	113.02、683.18、507.15、175.02	C_31_H_24_O_7_	Bulbocodioidins G	M	Phenanthrene
27	14.79	[M + C_6_H_7_O_6_]^-^	417.11938	0.67	113.02、85.03、121.03、175.02、241.09	C_15_H_14_O_3_	Coelonin	B/M	Phenanthrene
28	14.81	[M + C_12_H_15_O_12_]^-^	701.20776	−1.34	525.18、113.02、255.10、349.15	C_22_H_22_O_4_	2-(*p*-Hydroxybenzyl)-3’,5-dihydroxy-3-methoxybibenzyl	B/M	Bibenzyl
29	15.02	[M-H]^-^	491.15628	0.81	133.07、123.05、161.06、187.04、285.10	C_24_H_28_O_11_	Grammatophylloside A	B/M	Glycoside
30	15.02	[M + HCOO]^-^	771.27332	2.10	457.17、123.05、127.08、129.06、153.06	C_34_H_46_O_17_	Militarine	B/M	Glycosides
31	15.2	[M-H]^-^	137.02429	−0.88	93.03、137.02、108.02	C_7_H_6_O_3_	*p*-Hydroxybenzoic acid	B/M	Organic acid
32	15.2	[M + C_6_H_7_O_6_]^-^	415.10377	0.77	415.10、239.07、224.05、175.03、113.02	C_15_H_12_O_3_	2,7-Dihydroxy-4-methoxy-phenanthrene	M	Phenanthrene
33	15.47	[M + C_6_H_7_O_6_]^-^	419.13501	0.62	113.02、243.10、85.03、227.07	C_15_H_16_O_3_	Batatasin III	B/M	Bibenzyl

Note: B and M indicate Bingqiuzi and Maocigu, respectively.

In this study, we found that the glycosides obtained through spectrum–effect relationship analysis are converted into gastrodin and 2-isobutylmalic acid *in vivo*; phenanthrenes and bibenzyls can enter the bloodstream by binding to glucuronic acid; malic acid, citric acid, and batatasin III were not screened by spectrum–effect relationship analysis, but they are relatively abundant in medicinal materials and can enter the bloodstream. Existing literature has also reported their antitumor activities (Tong et al., 2021; Pinkhien et al., 2017). Therefore, 11 candidate active components were selected from two different commercial specifications of Shancigu, namely, monbarbatain A, 2,7-dihydroxy-1-(4-hydroxybenzyl)-4-methoxyphenanthrene, coelonin, blestriarene A, blestriarene B, 2-(*p*-hydroxybenzyl)-3′,5-dihydroxy-3-methoxybibenzyl, batatasin III, gastrodin, 2-isobutylmalic acid, malic acid, and citric acid. There was a significant difference in the content of these components between Bingqiuzi and Maocigu, indicating that Shancigu from different commercial specifications may exert antitumor effects through different proportions of the candidate active components.

### 3.5 Validation of the anti-liver cancer activities of potential active components *in vitro*

**FIGURE 4:**
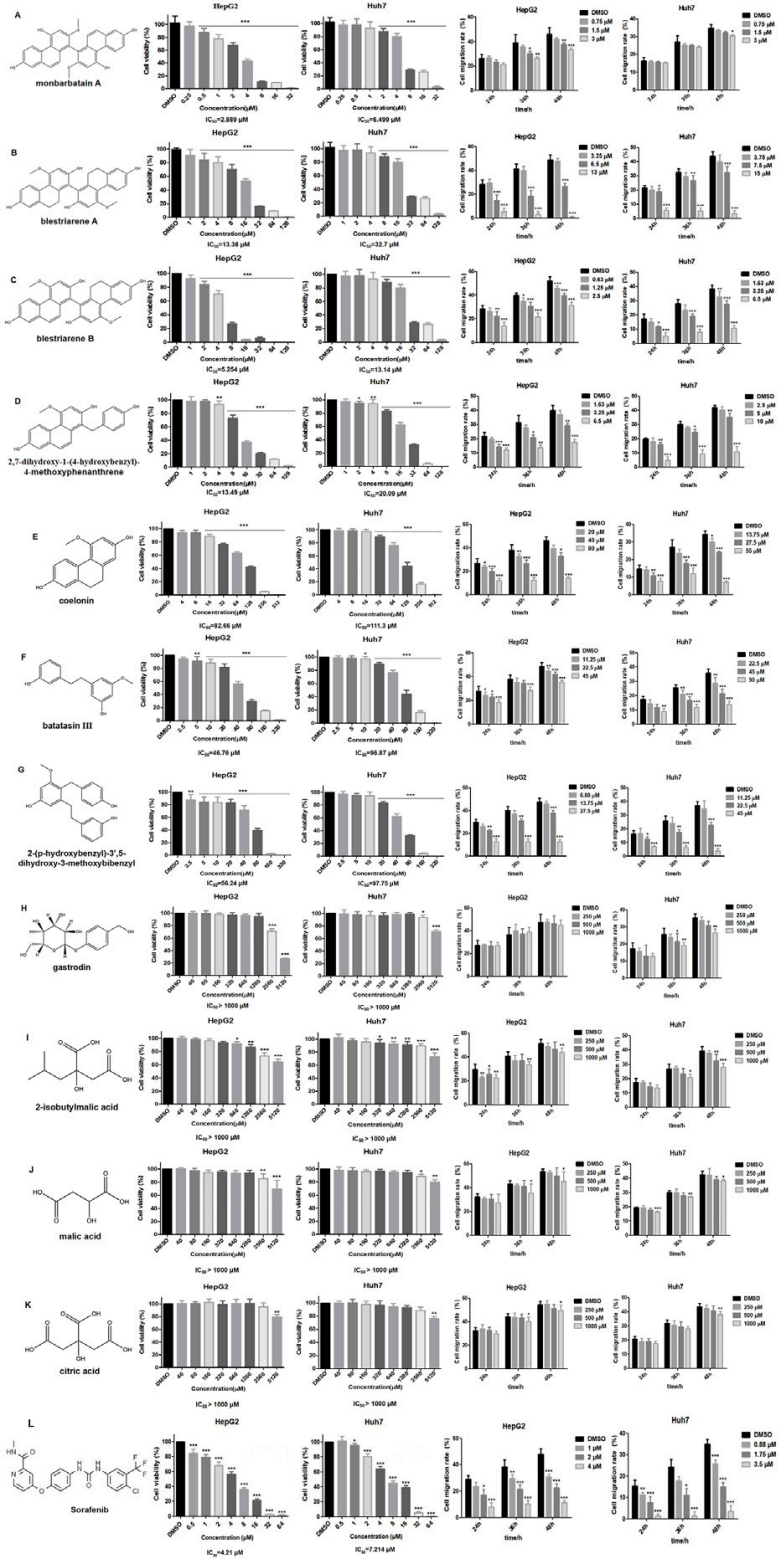
Effect of 11 potential active components and sorafenib on HepG2 and Huh7 cell viability and migration rate. **(A)** Monbarbatain A, **(B)** blestriarene A, **(C)** blestriarene B, **(D)** 2,7-dihydroxy-1-(4-hydroxybenzyl)-4-methoxyphenanthrene, **(E)** coelonin, **(F)** batatasin III, **(G)** 2-(p-hydroxybenzyl)-3’,5-dihydroxy- 3-methoxybibenzyl, **(H)** gastrodin, **(I)** 2-isobutylmalic acid, **(J)** malic acid, **(K)** citric acid, and **(L)** sorafenib. Data are presented as mean ± SD, *n* = 6; ^*^ *P* < 0.05, ^**^ *P* < 0.01, and ^***^ *P* < 0.001, treatment group vs control.

The effects of the 11 potential anti-liver cancer active components screened from Shancigu on HepG2 and Huh7 cell proliferation activity and migration ability were verified *in vitro* (Figures 4A–K). Sorafenib was used as the positive control (Figure 4L). The results indicated that all components could inhibit the proliferation of liver cancer cells to varying degrees. Among them, the phenanthrene components exhibited prominent anti-liver cancer activity; however, their contents were very low. For HepG2 cells, the IC_50_ value range was found to be 2.889 μM–82.66 μM; in particular, the IC_50_ value of monbarbatain A was 2.889 μM, which was lower than that of sorafenib at 4.21 μM, indicating that its inhibitory effect on HepG2 cell proliferation activity is stronger than that of sorafenib. The IC_50_ value of blestriarene B is 5.254 μM, which is similar in efficacy to that of sorafenib. For Huh7 cells, the IC_50_ value range was found to be 6.499 μM–111.3 μM, among which the IC_50_ value of monbarbatain A was 6.499 μM, lower than that of sorafenib at 7.214 μM, indicating that its inhibitory effect is stronger than that of sorafenib. The pharmacological effects of bibenzyl components were inferior to those of phenanthrene components. For HepG2 cells, the IC_50_ value range was 46.76 μM–56.24 μM, whereas for Huh7 cells, the IC_50_ value range was 92.75 μM–96.87 μM. The self-pharmacological effects of organic acids and gastrodin were not significant, but their contents were extremely high. They exerted certain antitumor activity at high concentrations and could play an auxiliary role, such as promoting specific biological processes by regulating the pH of the environment or providing suitable substrates, thereby enhancing the anti-liver cancer activity of phenanthrene and bibenzyl components.

All 11 potential anti-liver cancer active components could inhibit the migration ability of liver cancer cells to varying degrees, and this effect was dose- and time-dependent. Among them, the phenanthrene components exhibited the best pharmacological effects, showing significant inhibition on the migration of HepG2 cells within the range of 1.5 μM–80 μM. In particular, blestriarene B demonstrated the strongest migration-inhibiting activity. Similarly, within the range of 1.63 μM–55 μM, it also exhibits potent inhibitory effects on the migration of Huh7 cells, with blestriarene B again showing the best migration-suppressing efficacy. The inhibitory effect of bibenzyl components on cell migration is slightly weaker than that of phenanthrene components. For Huh7 cells, the migration inhibitory effect was exerted within the range of 11.25 μM–45 μM, whereas for Huh7 cells, it was effective within the range of 22.5 μM–90 μM. Gastrodin and organic acid components exhibited a certain degree of inhibitory effect on the migration ability of HepG2 and Huh7 cells at higher concentrations.

### 3.6 Validation of the anti-liver cancer pharmacological effects of potential effective component combinations *in vitro* and *in vivo*

**FIGURE 5:**
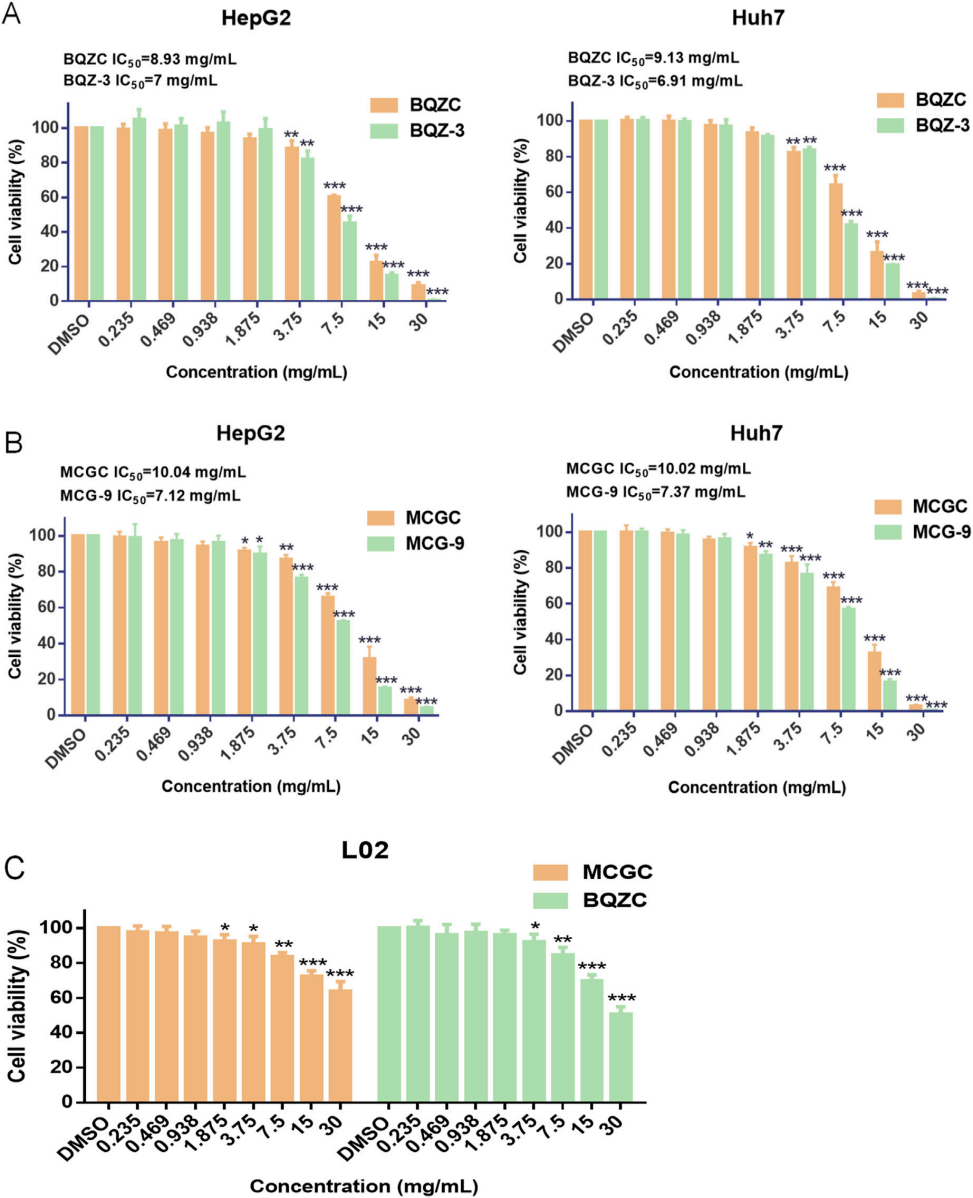
Anti-liver cancer activities of BQZC, BQZ-3, MCGC, and MCG-9 *in vitro*. **(A)** Effects of BQZC and BQZ-3 on the proliferation of HepG2 and Huh7 cells. **(B)** Effects of MCGC and MCG-9 on the proliferation of HepG2 and Huh7 cells. **(C)** Effects of BQZC and MCGC on the proliferation of L02 cells. Data are presented as mean ± SD, *n* = 6; ^*^ *P* < 0.05, ^**^ *P* < 0.01, and ^***^ *P* < 0.001, treatment group vs control.

Based on the content and proportion of the 11 effective components in batches BQZ-3 and MCG-9, which exhibited superior anti-liver cancer pharmacological effects *in vitro* (30 mg/mL crude medicinal botanical drugs) (Supplementary Tables S10, 11), the potential effective component combinations of Bingqiuzi and Maocigu for anti-liver cancer were obtained, and they are known as BQZC and MCGC, respectively. As the majority of the glycosidic components in Shancigu are hydrolyzed by digestive tract enzymes before entering the bloodstream as 2-isobutylmalic acid and gastrodin to exert drug effects, the contents of gastrodin and 2-isobutylmalic acid in the candidate effective component combinations were analyzed based on their contents after alkaline hydrolysis of the original medicinal materials (Liu et al., 2010). The effects of BQZ-3 and BQZC, and MCG-9 and MCGC, on the proliferation activity of HepG2 and Huh7 cells are shown in Figures 5A,B. There was no significant difference in the IC_50_ values between the effective component combinations of different commercial specifications of Shancigu and their respective extracts from the original medicinal materials at the same dosage of crude materials, indicating that BQZC and MCGC can represent the *in vitro* anti-liver cancer activity of the original medicinal materials to a certain extent. Compared with sorafenib, BQZC and MCGC exhibited selective cytotoxicity against liver cancer cells, whereas their toxicity toward L02 cells was lower (Figure 5C).

**FIGURE 6:**
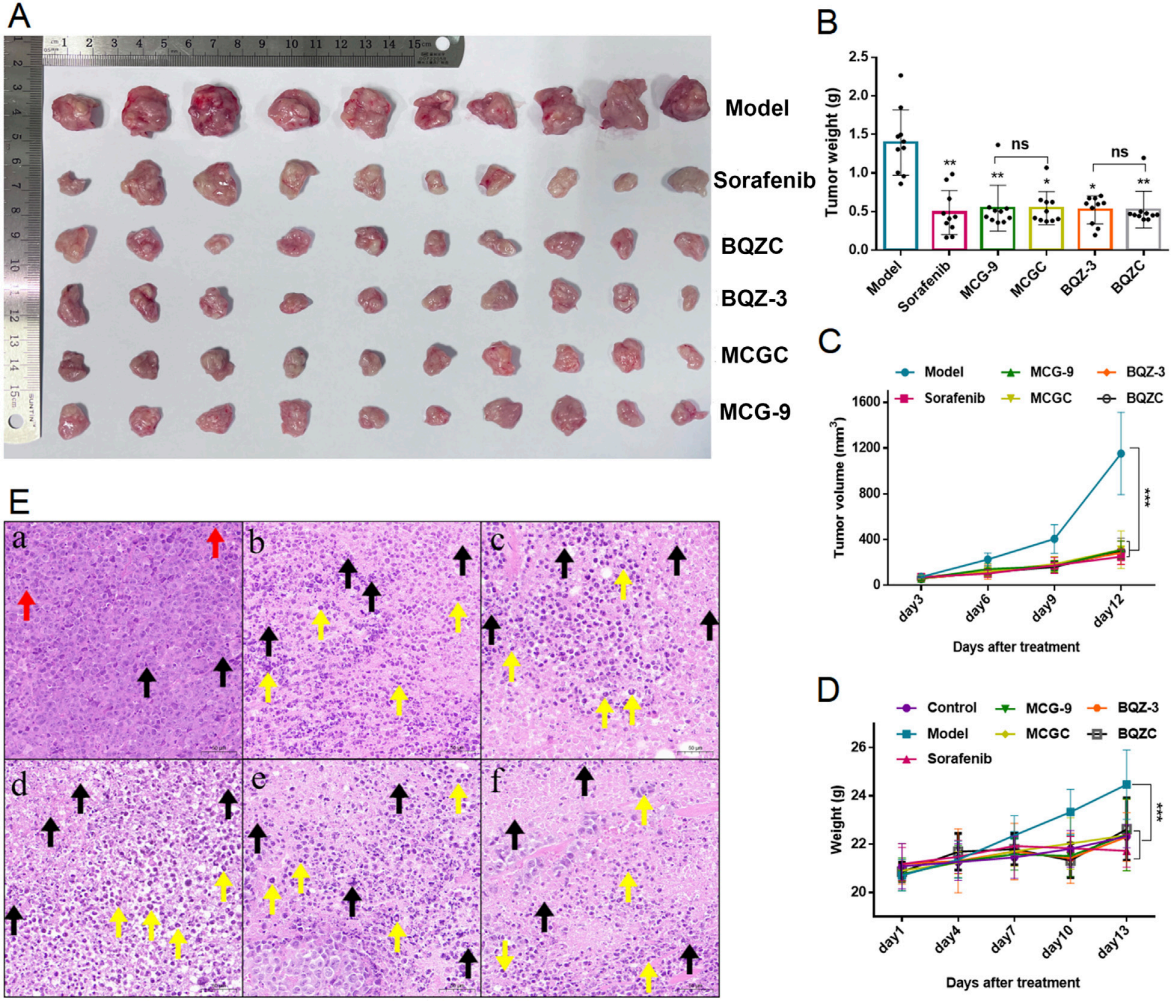
Effect of BQZ-3, BQZC, MCG-9, MCGC, and positive drugs on the growth of H22 xenografted tumors *in vivo*. **(A)** Tumor images. **(B)** Tumor weight. **(C)** Tumor growth curves. **(D)** The body weight was measured every 3 days. **(E)** Tumor histopathological morphology (HE staining, ×200, *n* = 3); (a) model group, (b) positive drug group, (c) BQZC group, (d) BQZ-3 group, (e) MCGC group, and (f) MCG-9 group, Note: the red arrow indicates the mitotic phase, the black arrow indicates necrotic cells, and the yellow arrow indicates the infiltration of inflammatory cells. Data are presented as mean ± SD; ^*^ *p* < 0.05, ^**^ *p* < 0.01, and ^***^ *p* < 0.001 vs model group; ns, not significant.

To further confirm the *in vivo* pharmacological effects, we used a xenograft model with H22 cells to evaluate the inhibitory effects of BQZC and MCGC on tumor growth. As shown in Figures 6A–C, BQZC, MCGC, and extracts of their raw medicinal materials significantly inhibited tumor growth by reducing the average volume and weight of the tumors (*P* < 0.01, *P* < 0.05, and *P* < 0.001). There was no significant difference between the BQZC and MCGC groups and their respective extract groups (*P* > 0.05). The tumor inhibition rates of each group are shown in Supplementary Table S12. As the administration time was prolonged, the model group showed a significant increase in body weight compared to the blank group (*P* < 0.001). Compared with those in the model group, the body weights of the mice in each treatment group decreased to varying degrees (*P* < 0.01, *P* < 0.001), indicating that tumor size was inhibited (Figure 6D). As shown in Figure 6E, the hematoxylin and eosin (H&E) staining images of each treatment group showed significant tumor necrosis, with a large number of inflammatory cells infiltrating the necrotic area.

### 3.7 Quantitative analysis of 11 components in effective component combinations

The results of the methodological validation confirmed the stability, accuracy, and reliability of the method. The RSD (%) of the peak area in the precision test, repeatability test, and stability test over 24 h were all less than 3.0%, and the RSD (%) of the retention time in these tests were all less than 0.3%. In addition, the recovery experiment showed RSDs of less than 2.00%. The standard curves of the 11 compounds exhibited good linearity (*R* ^*2*^ > 0.9990) for each reference standard within a certain concentration range. The results of the LOQ (Limit of Quantitation) and LOD (Limit of Detection) are listed in Table 3.

**TABLE 3:** Results of linear regression, LOQs, and LODs for the 11 effective components.

Component	Regression equation	*R* ^2^	Mass fraction (µg·mL^-1^)		
			Linearity range	LOD	LOQ
Malic acid	y = 18377351506.91570 x + 4416098399.33670	1	78.13–5000	0.07	0.25
Citric acid	y = 50673639.98068 x + 2292796323.41012	1	31.25–500	0.2	0.68
2-Isobutylmalic acid	y = 193270096.07830 x + 1003186896.70438	1	7.8125–125	0.023	0.076
Gastrodin	y = 42174535.45914 x + 676384816.86600	1	31.25–500	0.11	0.37
Batatasin III	y = 7279.75x+4244.39	0.9994	2.5–240	0.14	0.45
2-(p-Hydroxybenzyl)-3’, 5-dihydroxy-3-methoxybibenzyl	y = 6294.26x+2925.18	1	0.19–400	0.2	0.66
Coelonin	y = 18,466.83x-11001.29	0.9999	0.39–200	0.39	1.287
2,7-Dihydroxy-1-(4-hydroxybenzyl)-4-methoxyphenanthrene	y = 107,296.28x-342.35	1	0.02–12.5	0.02	0.05
Blestriarene A	y = 10,560.63x-2008.10	0.9999	0.26–33.34	0.08	0.26
Blestriarene B	y = 6445.91x-802.32	0.9995	0.26–16.67	0.007	0.021
Monbarbatain A	y = 269,712.81x-11700.24	0.9996	0.01–20	0.003	0.007

**FIGURE 7:**
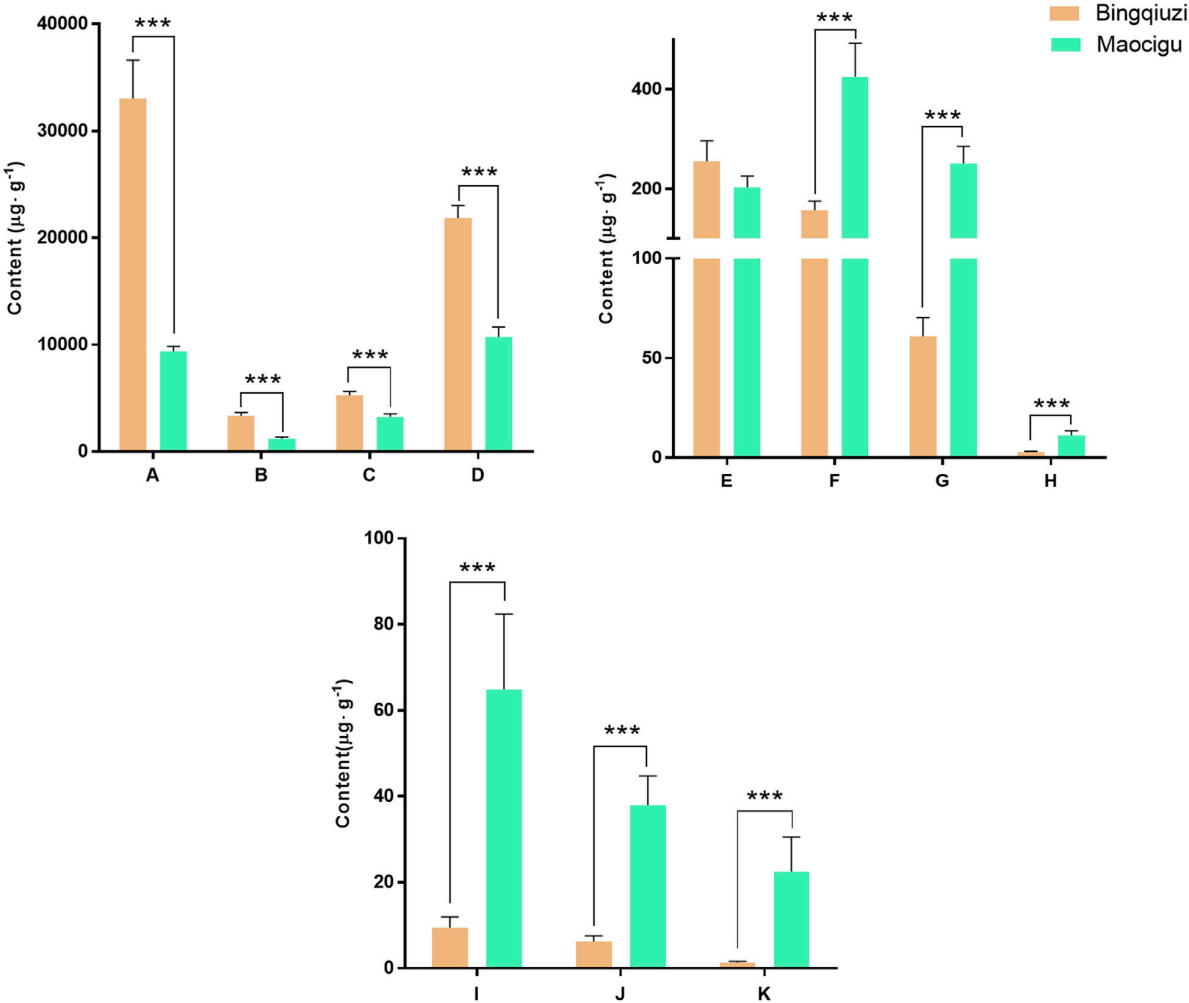
Determination of each effective component content of different commercial specifications of Shancigu (*n* = 12, *n* = 14, ^***^ *P* < 0.001). Note: A‐ malic acid, B‐ citric acid, C‐ 2-isobutylmalic acid, D‐ gastrodin, E‐ batatasin III, F‐ 2-(p-hydroxybenzyl)-3’,5-dihydroxy-3-methoxybibenzyl, G‐ coelonin, H‐ 2,7-dihydroxy-1-(4-hydroxybenzyl)-4-methoxyphenanthrene, I‐ blestriarene A, J‐ blestriarene B, and K‐ monbarbatain A.

The quantitative results of the 11 effective compounds in 14 batches of Bingqiuzi and 12 batches of Maocigu are shown in Supplementary Table S13. The content determination results of the 11 effective components in different commercial specifications of Shancigu are depicted in Figure 7. The contents of each component were observably different in samples from different commercial specifications. The contents of organic acids and glycosides were higher in Bingqiuzi, whereas the contents of phenanthrenes and bibenzyls were higher in Maocigu. These results implied that the quality of Shancigu depends on the interaction of multiple components. The anti-liver cancer ability of Bingqiuzi and Maocigu may be related to the total content of the 11 compounds. Therefore, it is important to use effective component combinations to comprehensively evaluate the anti-liver cancer quality of Shancigu samples.

### 3.8 Quality evaluation of Shancigu from different commercial specifications

**FIGURE 8:**
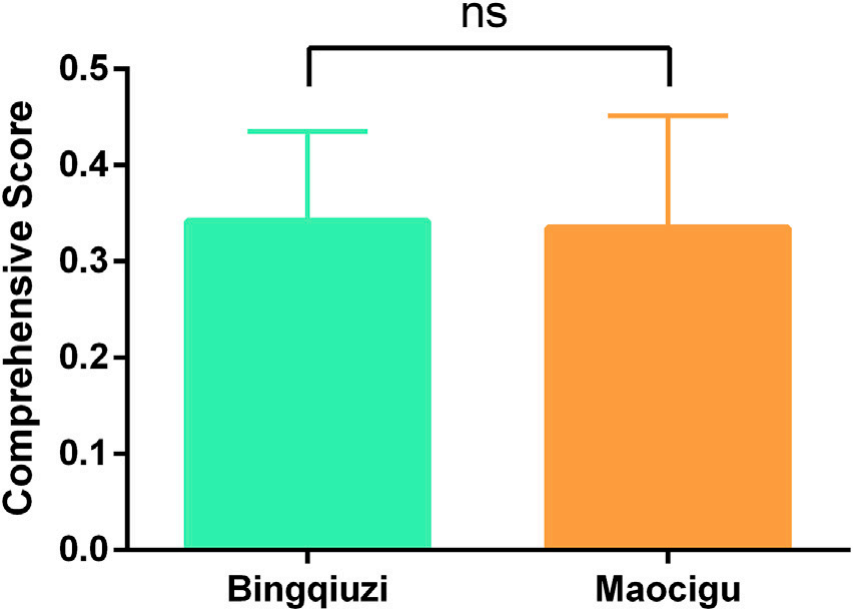
Quality comparison of different commercial specifications of Shancigu based on anti-liver cancer pharmacological effects. ns represents *P* > 0.05.

We calculated the comprehensive pharmacological effect indicators of 20 batches of Shancigu samples against liver cancer using the entropy weight method (Supplementary Table S14), and we evaluated the contribution of the 11 active components to this pharmacological effect indicator using GRA (Supplementary Table S15). Based on these results, TOPSIS was used to calculate the comprehensive quality scores of 26 batches of Shancigu from different commercial specifications, using the content of the 11 active components as indicators and the contribution of each component to the pharmacological effects as the weight coefficient (Supplementary Table S16) (Figure 8). From the perspective of anti-liver cancer pharmacological effects, there was no significant difference in the quality of Shancigu from different commercial specifications, which provides a basis for the combined use of Bingqiuzi and Maocigu as Shancigu.

### 3.9 Quality evaluation of Shancigu from different production areas

**FIGURE 9:**
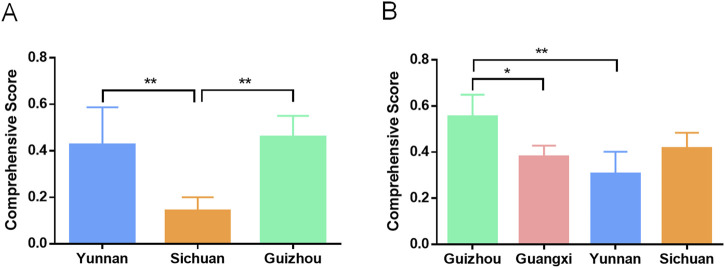
Quality comparison of Bingqiuzi **(A)** and Maocigu **(B)** from different regions based on anti-liver pharmacological effects. ^*^ *p* < 0.05 and ^**^ *p* < 0.01.

We distinguished the samples of Shancigu as either Bingqiuzi or Maocigu and separately evaluated the quality of Bingqiuzi and Maocigu from different production areas using the entropy weight method, GRA, and TOPSIS (Supplementary Table S17, 18) (Figure 9). The results indicated that for Bingqiuzi, the quality of medicinal materials from Guizhou and Yunnan was better, followed by those from Sichuan. For Maocigu, the medicinal materials produced in Guizhou and Sichuan were of better quality, followed by those from Guangxi and Yunnan.

## 4 Discussion

The selection of quality control indicators and the evaluation of quality for multisource Chinese medicinal materials have been central challenges in TCM quality control research. As stipulated by the *General Rules for Commercial Specifications and Grades of Chinese Medicinal Materials* (SB/T 11173-2016), different sources of the same TCM often circulate in the market under different commercial specifications, due to significant differences in their appearance and chemical composition, which lead to quality differences. For example, because of differences in characteristics from different sources, Curcuma Radix is classified as “Wenyujin,” “Huangsiyujin,” “Guiyujin,” and “Lvsiyujin,” with notable discrepancies in chemical composition content (Wang et al., 2022). Similarly, Euodiae Fructus is divided into small-flower Wuzhuyu, medium-flower Wuzhuyu, and large-flower Wuzhuyu according to the size of the fruit grain head, with diverse components and hepatotoxicity (Zhang et al., 2021). Multiple commercial specifications often imply poor chemical consistency, which has led to the fact that the quality control indicators for Shancigu are still not specified in the Chinese Pharmacopoeia, making it difficult to guide its quality evaluation and ensure the effectiveness and quality controllability of its clinical applications. The same situation applies to Yujin, Dahuang, and other medicinal materials. Therefore, taking Shancigu as an example, we attempted to establish a method for selecting quality control indicators and evaluating the quality of multi-commercial specification Chinese medicinal materials.

In previous studies on the differences in the chemical compositions of different commercial specifications of Shancigu, we had identified the differential markers between the two, but we had not yet clarified the similarities and differences in their pharmacodynamic substance basis (Hao et al., 2023). In this study, through spectrum–effect relationship analysis, serum pharmacochemistry, and *in vivo* and *in vitro* liver cancer models, the effective component combinations of different commercial specifications of Shancigu against liver cancer were screened and verified. It was clarified that different commercial specifications of Shancigu exert their pharmacological effects through different proportions of malic acid, citric acid, 2-isobutylmalic acid, gastrodin, batatasin III, 2-*p*-hydroxybenzyl-5,3′-dihydroxy-3-methoxybibenzyl, coelonin, 1-p-hydroxybenzyl-2,7-dihydroxy-4-methoxyphenanthrene, blestriarene A, blestriarene B, and monbarbatain A, totaling 11 components, thereby revealing their pharmacodynamic substance basis and exploring potential quality evaluation indicators. The contents of phenanthrenes and bibenzyls in Maocigu were higher than those in Bingqiuzi, whereas the contents of organic acids and gastrodin were higher in Bingqiuzi. Among them, phenanthrenes and bibenzyls exhibited excellent anti-liver cancer pharmacological effects but had relatively low contents, whereas organic acids and gastrodin showed poor pharmacological effects but had high contents. This ingredient-screening approach can effectively reflect the synergistic characteristics of multiple components in TCM, restoring the contribution of various components to pharmacological effects.

At present, research on the quality evaluation of Shancigu mostly adopts the method of fingerprint analysis combined with content determination (Xiao et al., 2023). However, these studies ignore the characteristics of multi-commercial specifications and use single indicators that are not related to pharmacological effects for evaluation, making it difficult to comprehensively assess the quality of Shancigu. In order to reflect the contribution of multiple components to pharmacological effects in quality evaluation methods and to distinguish the importance of different components, we used GRA to correlate the content of selected components with pharmacological effects and evaluate their pharmacological effect contributions. A quality evaluation method based on TOPSIS was established, using the contribution degree as the weight and the content of each component as the indicator. Subsequently, quality evaluations were conducted for Shancigu from different commercial specifications and production areas. In terms of different commercial specifications, there was no significant difference in anti-liver cancer quality between Bingqiuzi and Maocigu, demonstrating the rationality of both being used as Shancigu. Regarding different production areas, Bingqiuzi from Guizhou and Yunnan, along with Maocigu from Guizhou and Sichuan, exhibited better quality, providing a reference for the resource utilization of Shancigu. This quality evaluation method can not only assess the quality of TCM based on the content of multiple components but also reflect the differences in the contribution of each component to efficacy, making the quality evaluation results more scientific and reliable.

Due to differences in their origins, multisource TCMs exhibit variations across different sources, making it difficult to establish suitable evaluation criteria for unified assessment and analysis. Additionally, the large number of batches involved in research introduces further limitations. Traditional component analysis often focuses only on partial constituents, neglecting the holistic nature of TCM. Consequently, the quality evaluation methods established based on the content of single components fail to reflect the true efficacy of TCM. Therefore, it is recommended that research workers use techniques such as mass spectrometry to conduct comprehensive component analysis of botanical drugs from different sources to identify differences and similarities. By comparing their traditional therapeutic effects, modern pharmacological actions, and clinical applications, an appropriate efficacy evaluation model can be selected. Subsequently, *in vitro* efficacy assessment and spectrum–effect correlation methods can be adopted to efficiently and cost-effectively screen active constituents. Finally, by integrating these findings through data analysis techniques, a holistic quality control method based on multiple bioactive indicators can be established. This approach not only reflects the differences in the content of marker components but also distinguishes their contributions to the overall efficacy of TCM, making the quality evaluation results more accurate and scientifically robust.

## 5 Conclusion

To address the issue of lacking clear quality evaluation criteria and comprehensive quality assessment methods for different specifications of Shancigu, in this study, we screened and validated an anti-liver cancer effect-based component combination (consisting of 11 active components) through spectrum–effect relationship analysis, serum pharmacochemistry, and *in vitro*/*in vivo* pharmacological models. The contents of these 11 components were determined in 26 batches of Shancigu. Taking the contents of the 11 components of the effective component combinations against liver cancer as the index, the quality of Shancigu from different commercial specifications was evaluated using GRA and TOPSIS. The results demonstrated that there was no significant difference in quality between Bingqiuzi and Maocigu for the treatment of liver cancer. For Bingqiuzi, the quality of medicinal materials from Guizhou and Yunnan was better, whereas for Maocigu, those from Guizhou and Sichuan were of better quality. The developed methodology enables an in-depth assessment of Shancigu’s intrinsic quality and provides a basis for harnessing high-quality anticancer resources. This bioactivity-oriented, multicomponent, comprehensive quality evaluation system will provide a valuable reference for the quality control of other Chinese medicinal materials.

## Data Availability

The original contributions presented in the study are included in the article/Supplementary Material; further inquiries can be directed to the corresponding author.

## Abbreviations

BCA, bivariate correlation analysis; GRA, gray relational analysis; TCM, traditional Chinese medicine; TOPSIS, technique for order preference by similarity to ideal solution.
