# Supplementary material for: Establishment of a new approach for quality evaluation of Pseudobulbus Cremastrae seu Pleiones (Shancigu) based on multicomponent analysis and anti-liver cancer pharmacological effects
Source: Front Pharmacol. 2025 Jun 12;16:1544982. doi: 10.3389/fphar.2025.1544982 (PMC12198120; doi:10.3389/fphar.2025.1544982)
Supplement: Supplementary file 1 [file DataSheet1.docx]

Supplementary Material

**Fig. S1.** The effect of MCG-1 on the proliferation ability of HepG2 and Huh7 cells. HepG2 and Huh7 cells were treated with sample MCG-1 of various concentrations for 24h and 48h, and CCK-8 assay was performed to detect the viability of these cell lines. Data are mean±SD, *n*=6, ^*^*P<*0.05, ^**^*P*<0.01, ^***^*P*<0.001, Treatment group vs. Control.

**Fig. S2.** Total ion chromatograms (TICs) of serum samples from mice with liver cancer in negative ion mode. (A) blank serum, (B) model serum, (C) serum of Bingqiuzi and (D) serum of Maocigu.

**Table S1.** CPPP source information and yield of each batch.

**Table S2.** Results of similarity analysis of 14 Bingqiuzi samples and 12 Maocigu samples.

**Table S3.** The peak areas of 46 common peaks in HPLC fingerprint of 14 batches of Bingqiuzi.

**Table S4.** The peak areas of 42 common peaks in HPLC fingerprint of 12 batches of Maocigu.

**Table S5.** Results of anti-liver cancer efficacy indexes in vitro determination of 20 batches of Shancigu.

**Table S6.** Correlations coefficient and rank of GRA analysis of Bingqiuzi.

**Table S7.** Correlations coefficient and rank of GRA analysis of Maocigu.

**Table S8.** Correlations coefficient and rank of BCA analysis of Bingqiuzi.

**Table S9.** Correlations coefficient and rank of BCA analysis of Maocigu.

**Table S10.** The content and proportion of each active component in Bingqiuzi

**Table S11.** The content and proportion of each active component in Maocigu

**Table S12.** Comparison of tumor inhibition rates among different groups.

**Table S13.** Content determination of 11 effective components in 26 batches of Shancigu.

**Table S14.** Comprehensive pharmacodynamic indexes of 20 batches of different commercial specifications of Shancigu against liver cancer based on entropy weight method.

**Table S15.** Contribution and ranking of 11 effective components of Shancigu against liver cancer based on GRA.

**Table S16.** Results of TOPSIS analysis of 26 batches Shancigu samples.

**Table S17.** Results of TOPSIS analysis of 14 batches Bingqiuzi samples from different areas.

**Table S18.** Results of TOPSIS analysis of 12 batches Maocigu samples from different areas

**
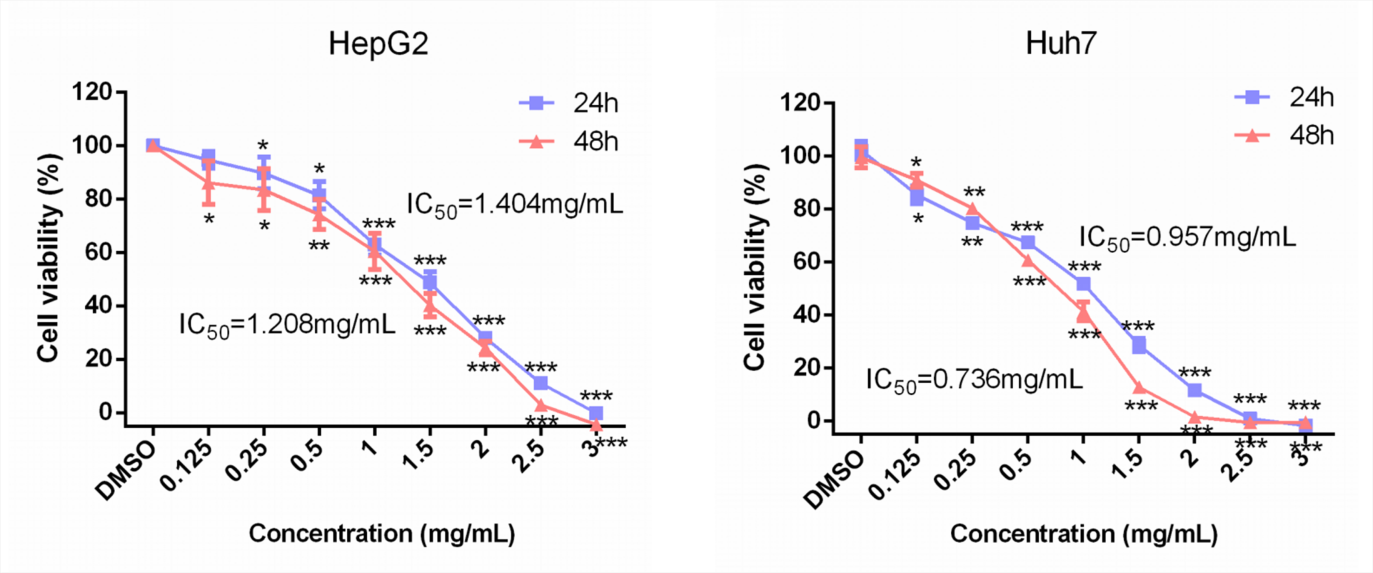
Fig. S1.** The effect of MCG-1 on the proliferation ability of HepG2 and Huh7 cells. HepG2 and Huh7 cells were treated with sample MCG-1 of various concentrations for 24h and 48h, and CCK-8 assay was performed to detect the viability of these cell lines. Data are mean±SD, *n*=6, ^*^*P<*0.05, ^**^*P*<0.01, ^***^*P*<0.001, Treatment group vs. Control.

**
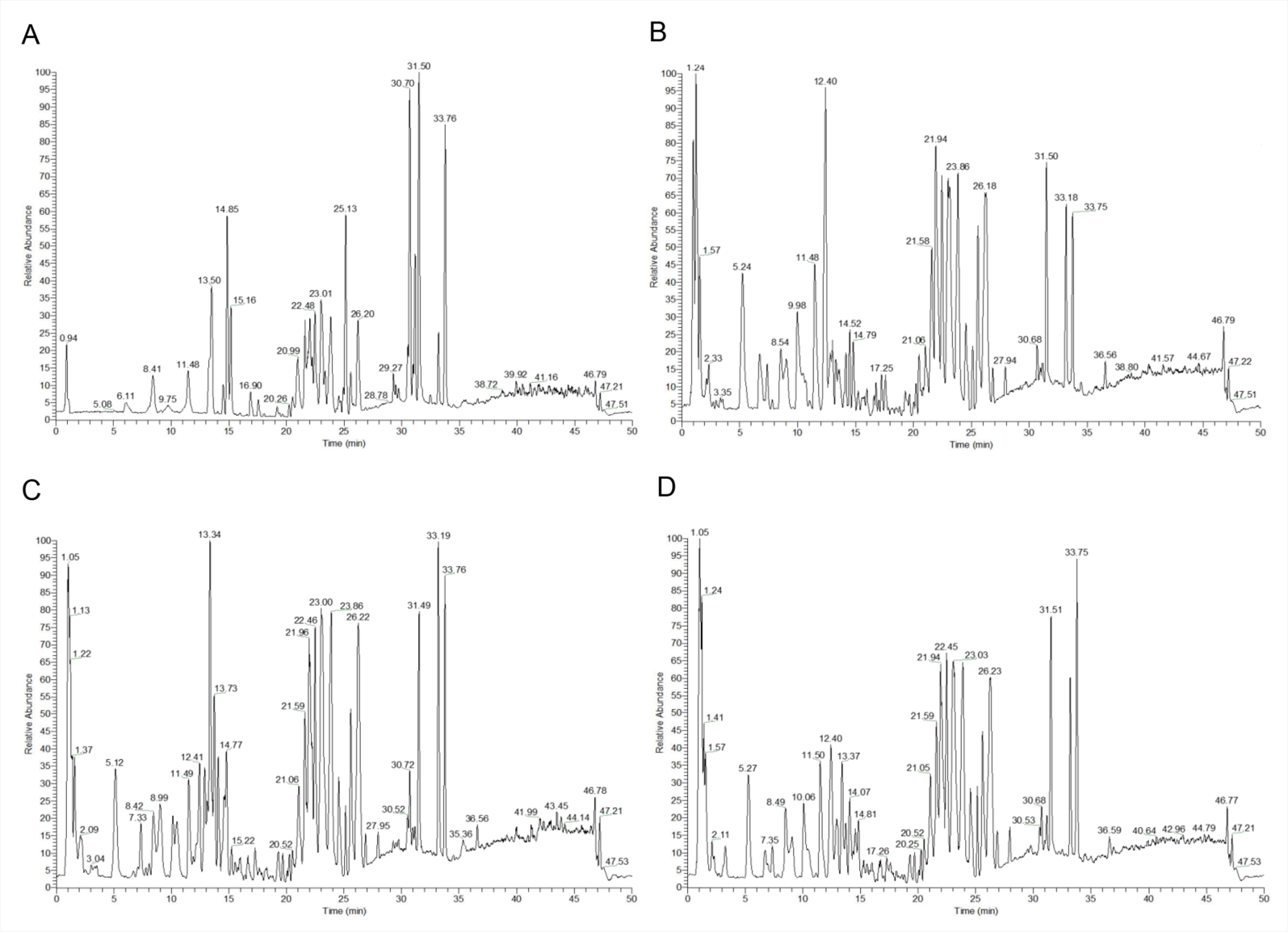
Fig. S2.** Total ion chromatograms (TICs) of serum samples from mice with liver cancer in negative ion mode. (A) blank serum, (B) model serum, (C) serum of Bingqiuzi and (D) serum of Maocigu.

**Table S1.** Shancigu source information and yield of each batch.

| **NO.** | **Name** | **Product specifications** | **Region** | **Plant origin** | **Yield (%)** |
| --- | --- | --- | --- | --- | --- |
| S1 | BQZ-1 | Bingqiuzi | Yunnan, Qujing | *Pleione bulbocodioides* (Franch.) Rolfe | 10.94% |
| S2 | BQZ-2 | Bingqiuzi | Yunnan, Qujing | *Pleione bulbocodioides* (Franch.) Rolfe | 13.25% |
| S3 | BQZ-3 | Bingqiuzi | Yunnan, Qujing | *Pleione bulbocodioides* (Franch.) Rolfe | 13.68% |
| S4 | BQZ-4 | Bingqiuzi | Guizhou, Bijie | *Pleione bulbocodioides* (Franch.) Rolfe | 13.83% |
| S5 | BQZ-5 | Bingqiuzi | Sichuan, Leshan | *Pleione bulbocodioides* (Franch.) Rolfe | 13.58% |
| S6 | BQZ-6 | Bingqiuzi | Sichuan, Leshan | *Pleione bulbocodioides* (Franch.) Rolfe | 17.17% |
| S7 | BQZ-7 | Bingqiuzi | Sichuan, Leshan | *Pleione bulbocodioides* (Franch.) Rolfe | 15.16% |
| S8 | BQZ-8 | Bingqiuzi | Yunnan, Lijiang | *Pleione bulbocodioides* (Franch.) Rolfe | 13.52% |
| S9 | BQZ-9 | Bingqiuzi | Yunnan, Lijiang | *Pleione bulbocodioides* (Franch.) Rolfe | 15.71% |
| S10 | BQZ-10 | Bingqiuzi | Yunnan, Lijiang | *Pleione bulbocodioides* (Franch.) Rolfe | 15.77% |
| S11 | BQZ-11 | Bingqiuzi | Guizhou, Qianxi | *Pleione yunnanensis* Rolfe | 17.03% |
| S12 | BQZ-12 | Bingqiuzi | Guizhou, Qianxi | *Pleione yunnanensis* Rolfe | 17.42% |
| S13 | BQZ-13 | Bingqiuzi | Guizhou, Qianxi | *Pleione yunnanensis* Rolfe | 18.67% |
| S14 | BQZ-14 | Bingqiuzi | Guizhou, Qianxi | *Pleione yunnanensis* Rolfe | 18.40% |
| S15 | MCG-1 | Maocigu | Guizhou, Zunyi | *Cremastra appendix* (D. Don) Makino | 9.29% |
| S16 | MCG-2 | Maocigu | Guizhou, Zunyi | *Cremastra appendix* (D. Don) Makino | 9.76% |
| S17 | MCG-3 | Maocigu | Guizhou, Zunyi | *Cremastra appendix* (D. Don) Makino | 9.29% |
| S18 | MCG-4 | Maocigu | Guangxi, Yulin | *Cremastra appendix* (D. Don) Makino | 6.50% |
| S19 | MCG-5 | Maocigu | Guangxi, Yulin | *Cremastra appendix* (D. Don) Makino | 7.62% |
| S20 | MCG-6 | Maocigu | Guangxi, Yulin | *Cremastra appendix* (D. Don) Makino | 7.94% |
| S21 | MCG-7 | Maocigu | Yunnan, Qujing | *Cremastra appendix* (D. Don) Makino | 7.67% |
| S22 | MCG-8 | Maocigu | Yunnan, Qujing | *Cremastra appendix* (D. Don) Makino | 10.54% |
| S23 | MCG-9 | Maocigu | Yunnan, Qujing | *Cremastra appendix* (D. Don) Makino | 11.17% |
| S24 | MCG-10 | Maocigu | Sichuan, Mabianshan | *Cremastra appendix* (D. Don) Makino | 11.07% |
| S25 | MCG-11 | Maocigu | Sichuan, Mabianshan | *Cremastra appendix* (D. Don) Makino | 9.31% |
| S26 | MCG-12 | Maocigu | Sichuan, Mabianshan | *Cremastra appendix* (D. Don) Makino | 12.49% |

**Table S2.** Results of similarity analysis of 14 Bingqiuzi samples and 12 Maocigu samples.

| **NO.** | **Similarity analysis** | **NO.** | **Similarity analysis** |
| --- | --- | --- | --- |
| BQZ-1 | 0.991 | BQZ-14 | 0.992 |
| BQZ-2 | 0.991 | MCG-1 | 0.969 |
| BQZ-3 | 0.991 | MCG-2 | 0.977 |
| BQZ-4 | 0.997 | MCG-3 | 0.996 |
| BQZ-5 | 0.990 | MCG-4 | 0.990 |
| BQZ-6 | 0.988 | MCG-5 | 0.982 |
| BQZ-7 | 0.987 | MCG-6 | 0.979 |
| BQZ-8 | 0.986 | MCG-7 | 0.981 |
| BQZ-9 | 0.997 | MCG-8 | 0.987 |
| BQZ-10 | 0.990 | MCG-9 | 0.990 |
| BQZ-11 | 0.991 | MCG-10 | 0.996 |
| BQZ-12 | 0.994 | MCG-11 | 0.996 |
| BQZ-13 | 0.992 | MCG-12 | 0.992 |

**Table S3.** The peak areas of 46 common peaks in HPLC fingerprint of 14 batches of Bingqiuzi.

| **NO.** | **RT/min** | **BQZ-1** | **BQZ-2** | **BQZ-3** | **BQZ-4** | **BQZ-5** | **BQZ-6** | **BQZ-7** | **BQZ-8** | **BQZ-9** | **BQZ-10** | **BQZ-11** | **BQZ-12** | **BQZ-13** | **BQZ-14** |
| --- | --- | --- | --- | --- | --- | --- | --- | --- | --- | --- | --- | --- | --- | --- | --- |
| 1 | 3.166 | 10174842 | 8694752 | 10416138 | 10515722 | 9578978 | 11666601 | 10413172 | 13132437 | 12866081 | 13375993 | 16000383 | 15064185 | 13942659 | 16547918 |
| 2 | 5.215 | 1777153 | 1461493 | 1771730 | 2025358 | 1731659 | 2429858 | 1619676 | 2953797 | 2428305 | 2733771 | 3814653 | 4279881 | 3456250 | 4480612 |
| 3 | 13.317 | 399663 | 222669 | 338778 | 151385 | 128222 | 97651 | 64416 | 34454 | 59877 | 49255 | 160784 | 193256 | 106811 | 183645 |
| 4 | 15.046 | 731824 | 256317 | 1046978 | 284094 | 421119 | 158878 | 241165 | 1023918 | 690618 | 628213 | 1378598 | 1477904 | 270114 | 1581954 |
| 5 | 15.361 | 12845936 | 17070664 | 18503956 | 17178052 | 13976614 | 19519630 | 15787807 | 15698333 | 19839162 | 19867286 | 14323872 | 15801869 | 15197803 | 17919952 |
| 6 | 16.176 | 647986 | 736151 | 879234 | 678497 | 666322 | 847069 | 729034 | 1577167 | 1383776 | 1397733 | 411566 | 454256 | 261585 | 464198 |
| 7 | 16.712 | 112809 | 16901 | 172670 | 85542 | 78904 | 97759 | 155049 | 558261 | 342235 | 382841 | 1594560 | 1569250 | 335293 | 2118756 |
| 8 | 16.999 | 47180 | 73667 | 53246 | 78490 | 33735 | 54983 | 31187 | 215732 | 178960 | 57161 | 37435 | 31931 | 119750 | 252344 |
| 9 | 22.071 | 226790 | 320056 | 433639 | 655725 | 244423 | 260883 | 243056 | 614054 | 752923 | 817330 | 92150 | 374597 | 558149 | 64423 |
| 10 | 23.003 | 22277886 | 29861358 | 32540192 | 32920982 | 22741966 | 31116900 | 22890004 | 41654560 | 42930732 | 43051484 | 35479880 | 36963500 | 41317616 | 37218208 |
| 11 | 24.904 | 62880 | 42866 | 66760 | 168422 | 20648 | 44812 | 8220 | 235811 | 239345 | 35494 | 97604 | 87146 | 293752 | 145320 |
| 12 | 25.405 | 260253 | 452461 | 344839 | 452220 | 173416 | 259719 | 195971 | 706954 | 541559 | 614048 | 647924 | 619693 | 731520 | 987333 |
| 13 | 25.845 | 580423 | 687641 | 1001214 | 2581580 | 1127569 | 994430 | 1417144 | 2907102 | 2679358 | 3441154 | 4960331 | 5280224 | 991502 | 6441964 |
| 14 | 30.869 | 88991 | 120274 | 113363 | 377225 | 96244 | 115460 | 107157 | 164201 | 219811 | 218929 | 362779 | 338692 | 203793 | 372473 |
| 15 | 31.754 | 463225 | 241655 | 650329 | 425320 | 179104 | 154506 | 263159 | 323656 | 300050 | 402309 | 737483 | 625860 | 261605 | 912649 |
| 16 | 32.188 | 38547 | 36314 | 20429 | 372556 | 140298 | 182596 | 178727 | 116611 | 193181 | 232080 | 203500 | 167453 | 97500 | 101353 |
| 17 | 33.15 | 33781 | 39160 | 49438 | 48479 | 29685 | 26653 | 37504 | 66043 | 73108 | 77177 | 74440 | 73147 | 44464 | 117118 |
| 18 | 34.541 | 45883 | 61466 | 100668 | 48499 | 28961 | 44846 | 104160 | 58058 | 42272 | 37636 | 69126 | 73133 | 129958 | 95600 |
| 19 | 35.941 | 2133036 | 3200777 | 3924004 | 590391 | 561985 | 890741 | 796324 | 572625 | 589454 | 704972 | 213520 | 225264 | 153133 | 219668 |
| 20 | 36.579 | 41651 | 93499 | 69274 | 96403 | 40242 | 83837 | 82538 | 128212 | 192574 | 132064 | 36938 | 24790 | 37354 | 39482 |
| 21 | 36.892 | 89932 | 55582 | 143889 | 98660 | 51602 | 56117 | 71691 | 64546 | 48654 | 95775 | 211927 | 154691 | 50578 | 181421 |
| 22 | 37.413 | 141252 | 353129 | 216298 | 1004308 | 334881 | 647333 | 427499 | 862979 | 1507507 | 1012482 | 429445 | 514409 | 193891 | 547982 |
| 23 | 37.823 | 578851 | 906400 | 696242 | 3443668 | 1675302 | 2271064 | 1620533 | 4608339 | 3773180 | 4846137 | 1563411 | 1488652 | 1092179 | 1751942 |
| 24 | 38.215 | 329972 | 522904 | 503553 | 448773 | 520432 | 958646 | 756893 | 846556 | 996049 | 896919 | 237909 | 318147 | 148190 | 375883 |
| 25 | 38.812 | 1326323 | 2055269 | 1977164 | 2023525 | 2288089 | 3841755 | 3093447 | 3170856 | 4083236 | 3321520 | 1147157 | 1554532 | 1076670 | 1882055 |
| 26 | 39.243 | 514473 | 755261 | 719992 | 2107253 | 1337350 | 1833565 | 1331990 | 3549987 | 2852842 | 3612061 | 978442 | 1033750 | 769785 | 1212902 |
| 27 | 39.857 | 2046438 | 2316060 | 2862400 | 1067555 | 1219151 | 1321418 | 1222079 | 153254 | 233185 | 825582 | 776478 | 1017382 | 1210390 | 938097 |
| 28 | 40.486 | 30546 | 36618 | 29074 | 70816 | 25942 | 40470 | 32682 | 92898 | 87142 | 89364 | 74382 | 49182 | 78390 | 52491 |
| 29 | 41.826 | 36090 | 16048 | 65675 | 31112 | 22278 | 33202 | 32725 | 38858 | 51776 | 68101 | 150808 | 116664 | 39822 | 162639 |
| 30 | 41.251 | 42262 | 39157 | 63588 | 32204 | 57748 | 14159 | 71387 | 103009 | 87009 | 88519 | 60846 | 67931 | 73686 | 86010 |
| 31 | 43.19 | 2989723 | 5453135 | 4948291 | 4505999 | 1658731 | 2025599 | 2899712 | 13436201 | 9211179 | 14134307 | 5547568 | 6310602 | 7006226 | 9029365 |
| 32 | 43.784 | 541284 | 686524 | 864519 | 220210 | 305545 | 340352 | 407526 | 236431 | 221789 | 352382 | 466591 | 588159 | 172473 | 677475 |
| 33 | 45.364 | 211448 | 77918 | 331205 | 86974 | 58091 | 65307 | 122746 | 165204 | 117295 | 218546 | 841441 | 979315 | 101757 | 1103733 |
| 34 | 47.01 | 59090 | 9016 | 57187 | 75020 | 31523 | 65432 | 49471 | 48143 | 121281 | 31126 | 96147 | 59812 | 118849 | 88786 |
| 35 | 50.453 | 53637 | 78488 | 52322 | 59765 | 73907 | 147922 | 86219 | 102398 | 102688 | 137283 | 190910 | 241142 | 188066 | 253546 |
| 36 | 50.908 | 749260 | 731520 | 1008118 | 571645 | 283897 | 440001 | 503814 | 1178318 | 878595 | 1235886 | 938870 | 1013714 | 735380 | 1294044 |
| 37 | 51.696 | 412976 | 585098 | 577513 | 422874 | 257934 | 319024 | 349255 | 926073 | 710458 | 927483 | 899593 | 1072075 | 590034 | 1245018 |
| 38 | 52.676 | 73023 | 15017 | 106085 | 16161 | 13355 | 11415 | 22557 | 13625 | 16197 | 19931 | 32452 | 28219 | 16067 | 41831 |
| 39 | 53.861 | 46823 | 8249 | 68872 | 12633 | 7240 | 12798 | 24373 | 18024 | 20725 | 20066 | 31890 | 28699 | 13914 | 45172 |
| 40 | 54.773 | 132885 | 144484 | 183968 | 102350 | 68085 | 69963 | 94170 | 204440 | 195063 | 196011 | 167006 | 181954 | 235820 | 244880 |
| 41 | 55.571 | 183535 | 186761 | 229099 | 164548 | 138359 | 150732 | 195556 | 292681 | 289940 | 300049 | 200344 | 235246 | 146871 | 303264 |
| 42 | 57.856 | 136150 | 136836 | 163532 | 99648 | 90112 | 86846 | 126332 | 211522 | 181243 | 213088 | 118908 | 147000 | 73189 | 195501 |
| 43 | 60.74 | 244721 | 152360 | 387214 | 161482 | 137918 | 138428 | 238214 | 315289 | 292002 | 359741 | 256645 | 312116 | 249626 | 379938 |
| 44 | 62.55 | 83800 | 51704 | 161074 | 53640 | 22867 | 17460 | 37988 | 123701 | 83506 | 100069 | 70934 | 95464 | 72940 | 126427 |
| 45 | 65.259 | 438048 | 327196 | 506179 | 679997 | 195877 | 106358 | 264584 | 779610 | 655419 | 1195812 | 336535 | 339000 | 164261 | 450285 |
| 46 | 71.278 | 39127 | 27970 | 51261 | 52951 | 17904 | 10624 | 25222 | 71018 | 52524 | 126953 | 25428 | 26209 | 9150 | 39875 |

**Table S4.** The peak areas of 42 common peaks in HPLC fingerprint of 12 batches of Maocigu.

| **NO.** | **RT/min** | **MCG-1** | **MCG-2** | **MCG-3** | **MCG-4** | **MCG-5** | **MCG-6** | **MCG-7** | **MCG-8** | **MCG-9** | **MCG-10** | **MCG-11** | **MCG-12** |
| --- | --- | --- | --- | --- | --- | --- | --- | --- | --- | --- | --- | --- | --- |
| 1 | 3.188 | 11162926 | 8618741 | 9078809 | 5360109 | 5065781 | 4573492 | 8119631 | 7733295 | 8440147 | 7701065 | 6884583 | 7286442 |
| 2 | 5.228 | 1956262 | 1246623 | 1907953 | 1093889 | 766930.8 | 1131091 | 1655793 | 1525971 | 1862147 | 1748031 | 1088341 | 1762558 |
| 3 | 14.295 | 213273.4 | 168159 | 237893.6 | 122736.7 | 132878 | 144836.6 | 187254.6 | 251810.2 | 233932.2 | 234674.8 | 235214.2 | 199015 |
| 4 | 14.725 | 60030.66 | 53063.12 | 80237.2 | 41351.02 | 31506.95 | 43690.26 | 76833.94 | 133799.9 | 116619.2 | 81827.87 | 63317.26 | 78538.34 |
| 5 | 15.13 | 3359581 | 3049404 | 4333678 | 3076402 | 3406394 | 3688171 | 4534287 | 5516182 | 4948729 | 4534155 | 4213242 | 4611863 |
| 6 | 15.445 | 490033.9 | 473539.9 | 610897.6 | 2257509 | 1856713 | 2357915 | 836378.9 | 1078193 | 944961.1 | 747999.7 | 698986 | 470539.3 |
| 7 | 16.231 | 909414 | 965385.8 | 593721.8 | 870216.3 | 1029715 | 951230.8 | 300769.6 | 262474.1 | 553018.5 | 735917.7 | 586417.2 | 823872.9 |
| 8 | 16.771 | 861850.9 | 861559.2 | 885913.1 | 622714.3 | 748093 | 721549.6 | 464866.8 | 555078.1 | 628312.5 | 965762.3 | 867452.4 | 935620.5 |
| 9 | 17.963 | 100777 | 76457.45 | 90766.69 | 46440.94 | 69020.32 | 92655.7 | 112420.5 | 146361.1 | 129657.1 | 115466.6 | 101166.9 | 106617.9 |
| 10 | 18.255 | 144908.2 | 91727.13 | 515999 | 752650.4 | 205969 | 243542.2 | 184827.4 | 272968.1 | 235681.9 | 227148 | 182585.5 | 187317.5 |
| 11 | 19.538 | 43622.63 | 20075.7 | 20230.93 | 62298.65 | 62951.92 | 73064.81 | 75811.47 | 110499.9 | 57768.55 | 100941.4 | 38851.46 | 102001.5 |
| 12 | 21.876 | 135383.5 | 125679.6 | 154391 | 344656 | 150554.7 | 157979.1 | 271180.7 | 277896.6 | 320777.2 | 208759.8 | 146362.9 | 218548 |
| 13 | 22.22 | 314007.8 | 290355.3 | 385217.4 | 203322.5 | 196302.4 | 229740.2 | 711116.5 | 775535.1 | 815092.1 | 436617.5 | 255492.4 | 463586.6 |
| 14 | 23.134 | 14041626 | 11585630 | 16956602 | 13479720 | 13354839 | 14027640 | 20341234 | 22575534 | 21973976 | 18404476 | 16055890 | 17893540 |
| 15 | 24.565 | 5920804 | 4446683 | 7088456 | 3740222 | 4146687 | 4911532 | 6717375 | 7492425 | 8125984 | 8542842 | 7349575 | 8258449 |
| 16 | 25.555 | 130749.3 | 152500.2 | 251135.1 | 159171.5 | 373158 | 293787.6 | 153869.8 | 319148.3 | 277078.4 | 452030.2 | 395044.7 | 473956.9 |
| 17 | 31.021 | 67317.99 | 60301.51 | 48476.48 | 66845.19 | 61545.03 | 60875.19 | 158032 | 112533.7 | 115072.5 | 182245.8 | 168935.5 | 193862.5 |
| 18 | 31.918 | 971335.9 | 1264172 | 1643450 | 918915.9 | 1782741 | 1605590 | 372534.9 | 472249 | 465752 | 1133026 | 1107004 | 1295317 |
| 19 | 34.656 | 13413.05 | 23803.45 | 88156.31 | 39616.45 | 107966.8 | 67652.45 | 54896.38 | 80139.48 | 47512.27 | 41164.93 | 37286.78 | 28937.87 |
| 20 | 35.071 | 68883.92 | 57368.69 | 114889.5 | 43253.51 | 40216.73 | 22765.23 | 67826.66 | 85646.78 | 54902.67 | 56261.11 | 47328.34 | 71030.25 |
| 21 | 36.618 | 23527.07 | 35524.43 | 68807.12 | 22021.94 | 32931.37 | 26530.2 | 10413.43 | 23035.56 | 23035.55 | 59057.98 | 26600.63 | 56846.36 |
| 22 | 37.035 | 48379.85 | 38174.22 | 50319.78 | 265909.1 | 245958.8 | 308803.8 | 34788.59 | 106646.3 | 43747.39 | 51564.43 | 35622.71 | 40144.17 |
| 23 | 39.408 | 51707.93 | 46559.78 | 54418.85 | 68214.22 | 70548.47 | 66205.93 | 81788.71 | 112392.2 | 108843.8 | 66054.44 | 78846.32 | 30765.08 |
| 24 | 40.054 | 42000.66 | 31896.28 | 54165.42 | 65880.8 | 45594.4 | 58698.81 | 66193.55 | 75005.38 | 57407 | 49429.13 | 45124.19 | 45860.56 |
| 25 | 40.641 | 55536.56 | 49777.49 | 78369.4 | 49282.03 | 54374.43 | 47869.01 | 91478.7 | 118742.8 | 99143.86 | 73134.33 | 71562.1 | 62347.94 |
| 26 | 40.897 | 121102.1 | 104880.5 | 142731.3 | 100010.5 | 105707.5 | 88875.7 | 131213.1 | 186190 | 112975.9 | 164388.2 | 127058.1 | 116784.9 |
| 27 | 41.974 | 238896.9 | 228469.4 | 290925.4 | 137518.6 | 137218.7 | 162767.9 | 110290.2 | 153656.7 | 108292.5 | 169730.9 | 132341.9 | 161195.4 |
| 28 | 43.336 | 2701953 | 4509374 | 5390066 | 4289533 | 6151096 | 6525661 | 1267072 | 2988513 | 2759457 | 6629424 | 5618608 | 7397015 |
| 29 | 45.506 | 521083.7 | 478171.7 | 541503.5 | 295519.7 | 267546.4 | 270326.7 | 105459 | 77528.38 | 95965.85 | 368595.7 | 258799.3 | 402851.7 |
| 30 | 47.187 | 323965.4 | 307058.1 | 445839.8 | 100276.4 | 111874.4 | 119323.1 | 88536.9 | 74015.73 | 93160.04 | 127246.6 | 118984.5 | 106294.3 |
| 31 | 48.49 | 130447.7 | 179062.8 | 231464.2 | 39617.97 | 33147.17 | 69768.48 | 20983.44 | 14520.29 | 30258.28 | 51723.45 | 34359.02 | 95401.56 |
| 32 | 50.6 | 369934.4 | 317061.5 | 394897.8 | 138565.9 | 202161.4 | 138188 | 250825.5 | 312156.1 | 204301.7 | 473323.5 | 308623.3 | 369123.4 |
| 33 | 51.09 | 1475550 | 1645911 | 2035430 | 678133.3 | 761267 | 880782.3 | 574369.8 | 392050.9 | 822181.5 | 1267877 | 1001096 | 1343848 |
| 34 | 51.858 | 515006.6 | 658203.9 | 758542.9 | 367055.5 | 396847.7 | 408656.6 | 330365.6 | 429522 | 477853.8 | 782602.3 | 637410.8 | 751746.6 |
| 35 | 52.842 | 401572.1 | 322670.4 | 573423.3 | 179256.1 | 212641.5 | 250232.4 | 126550.6 | 120306.1 | 179357.2 | 614782.3 | 630241.4 | 973769.1 |
| 36 | 53.997 | 371945 | 313211.4 | 543826.4 | 154925.2 | 157107.6 | 199490.7 | 140696.5 | 148818.8 | 212406.4 | 182423.1 | 129919 | 195881 |
| 37 | 55.696 | 364717.8 | 322370.1 | 542255.4 | 115431.3 | 118494.1 | 157707.7 | 140105.1 | 113868.8 | 169400.3 | 250046.3 | 128418.4 | 291484.6 |
| 38 | 57.99 | 190535.2 | 249717.2 | 290445.2 | 97091.91 | 84533.45 | 117038.5 | 72156.34 | 54355.52 | 88800.59 | 296666 | 168616.5 | 312406.7 |
| 39 | 60.841 | 338668.6 | 289338.7 | 469092.4 | 223644.8 | 246236.8 | 329896 | 81454.28 | 86317.96 | 151116.9 | 194536.5 | 115418.3 | 289250.1 |
| 40 | 67.935 | 69820.59 | 162235.2 | 86476.06 | 80501.3 | 52973.04 | 36803.81 | 33528.06 | 18786.87 | 36822.47 | 28106.14 | 19511.7 | 41606.42 |
| 41 | 77.109 | 92689.89 | 64712.98 | 87050.11 | 125932.6 | 177939.6 | 217029.7 | 154555.4 | 221475 | 173654.9 | 223046.1 | 116423.9 | 285374.9 |
| 42 | 77.704 | 139350 | 95898.39 | 93975.97 | 452541.4 | 357282.6 | 403489.6 | 140202.9 | 137600.3 | 93462.75 | 126531.1 | 98337.43 | 203083.4 |

**Table S5.** Results of anti-liver cancer efficacy indexes in vitro determination of 20 batches of Shancigu

| **Group** | **proliferative vitality** | | **migration vitality** | |
| --- | --- | --- | --- | --- |
|  | **HepG2** | **Huh7** | **HepG2** | **Huh7** |
| BQZ-1 | 64.44±4.20^***^ | 53.11±4.92^***^ | 25.79±3.48^***^ | 25.50±2.44^***^ |
| BQZ-2 | 96.16±4.17 | 63.31±3.25^***^ | 13.01±2.34^***^ | 26.28±4.46^***^ |
| BQZ-3 | 39.16±1.23^***^ | 11.40±1.00^***^ | 11.11±2.02^***^ | 22.50±3.11^***^ |
| BQZ-4 | 79.24±2.44^***^ | 75.52±3.71^***^ | 21.14±1.32^***^ | 28.41±3.46^***^ |
| BQZ-5 | 38.12±3.20^***^ | 24.96±1.57^***^ | 3.17±1.05^***^ | 12.54±1.86^***^ |
| BQZ-6 | 58.20±4.12^***^ | 60.98±1.86^***^ | 13.63±1.70^***^ | 26.67±7.91^***^ |
| BQZ-7 | 62.90±5.85^***^ | 50.54±2.08^***^ | 9.79±2.27^***^ | 15.99±4.97^***^ |
| BQZ-8 | 70.36±9.93^***^ | 63.63±5.63^***^ | 20.34±1.76^***^ | 30.00±2.80^***^ |
| BQZ-9 | 58.46±3.41^***^ | 70.74±2.85^***^ | 10.49±2.25^***^ | 33.12±3.72 |
| BQZ-10 | 74.17±2.85^***^ | 62.77±3.19^***^ | 11.01±1.33^***^ | 28.46±5.27^***^ |
| MCG-1 | 58.24±2.93^***^ | 61.41±3.57^***^ | 18.95±2.57^***^ | 20.01±1.76^***^ |
| MCG-4 | 82.85±4.46^***^ | 77.58±4.58^***^ | 21.53±0.89^***^ | 28.88±3.96^***^ |
| MCG-5 | 94.30±5.40^*^ | 60.75±2.71^***^ | 19.22±2.79^***^ | 28.50±4.45^***^ |
| MCG-6 | 89.57±4.03^***^ | 58.19±1.31^***^ | 17.38±3.24^***^ | 27.80±4.19^***^ |
| MCG-7 | 74.88±2.93^***^ | 75.61±2.55^***^ | 21.80±2.99^***^ | 24.34±2.86^***^ |
| MCG-8 | 91.95±3.93^**^ | 72.17±3.71^***^ | 9.53±1.44^***^ | 32.11±4.31^**^ |
| MCG-9 | 53.46±3.01^***^ | 51.04±1.12^***^ | 0.28±0.18^***^ | 27.43±3.82^***^ |
| MCG-10 | 55.75±3.42^***^ | 64.49±4.31^***^ | 20.69±1.27^***^ | 27.27±3.67^***^ |
| MCG-11 | 74.26±4.80^***^ | 62.42±3.24^***^ | 14.72±1.46^***^ | 28.08±5.14^***^ |
| MCG-12 | 58.81±2.95^***^ | 58.56±2.47^***^ | 15.09±2.37^***^ | 38.43±7.36 |

**Table S6.** Correlations coefficient and rank of GRA analysis of Bingqiuzi.

| NO. | Peak | Components | HepG2  proliferation inhibition | | | HepG2  migration inhibition | | | Huh7  proliferation inhibition | | | Huh7  migration inhibition | |
| --- | --- | --- | --- | --- | --- | --- | --- | --- | --- | --- | --- | --- | --- |
|  |  |  | Rank | Correlation coefficient | Rank | | Correlation coefficient | Rank | | Correlation coefficient | Rank | | Correlation coefficient |
| 1 | b1 | gastrodin | 3 | 0.761 | 12 | | 0.726 | 22 | | 0.665 | 15 | | 0.665 |
| 2 | b2 | 2-isobutylmalic acid | 14 | 0.715 | 23 | | 0.695 | 30 | | 0.629 | 27 | | 0.628 |
| 3 | b3 | Unknow | 27 | 0.656 | 32 | | 0.659 | 7 | | 0.743 | 7 | | 0.699 |
| 4 | b4 | loroglossin | 8 | 0.739 | 17 | | 0.714 | 11 | | 0.719 | 22 | | 0.654 |
| 5 | b5 | dactylorhin A | 9 | 0.734 | 1 | | 0.82 | 19 | | 0.682 | 5 | | 0.704 |
| 6 | b6 | gymnoside | 31 | 0.636 | 24 | | 0.695 | 23 | | 0.654 | 30 | | 0.616 |
| 7 | b7 | gymnoside II | 12 | 0.719 | 14 | | 0.72 | 20 | | 0.672 | 20 | | 0.654 |
| 8 | b9 | Unknow | 33 | 0.616 | 31 | | 0.672 | 34 | | 0.612 | 33 | | 0.6 |
| 9 | b10 | militarine | 28 | 0.656 | 6 | | 0.743 | 26 | | 0.639 | 14 | | 0.666 |
| 10 | b12 | Unknow | 32 | 0.635 | 18 | | 0.711 | 33 | | 0.627 | 26 | | 0.637 |
| 11 | b13 | shancigusin H | 26 | 0.66 | 27 | | 0.686 | 32 | | 0.628 | 34 | | 0.6 |
| 12 | b14 | Unknow | 30 | 0.639 | 26 | | 0.693 | 27 | | 0.636 | 32 | | 0.605 |
| 13 | b15 | coelonin | 13 | 0.717 | 28 | | 0.686 | 5 | | 0.751 | 17 | | 0.663 |
| 14 | b16 | Unknow | 4 | 0.759 | 4 | | 0.778 | 17 | | 0.687 | 25 | | 0.65 |
| 15 | b19 | Unknow | 34 | 0.612 | 34 | | 0.608 | 9 | | 0.732 | 18 | | 0.663 |
| 16 | b22 | Unknow | 25 | 0.666 | 16 | | 0.719 | 25 | | 0.645 | 28 | | 0.625 |
| 17 | b23 | gymnoside V | 23 | 0.672 | 19 | | 0.708 | 29 | | 0.631 | 31 | | 0.613 |
| 18 | b24 | Unknow | 16 | 0.713 | 3 | | 0.789 | 14 | | 0.694 | 16 | | 0.664 |
| 19 | b25 | gymnoside VI | 10 | 0.731 | 2 | | 0.799 | 13 | | 0.694 | 11 | | 0.679 |
| 20 | b26 | gymnoside IV | 21 | 0.686 | 13 | | 0.721 | 28 | | 0.635 | 29 | | 0.621 |
| 21 | b27 | gymnoside V isomer | 6 | 0.756 | 11 | | 0.726 | 2 | | 0.789 | 1 | | 0.773 |
| 22 | b31 | batatasin III | 29 | 0.642 | 21 | | 0.701 | 31 | | 0.628 | 24 | | 0.651 |
| 23 | b32 | Unknow | 18 | 0.698 | 29 | | 0.678 | 1 | | 0.821 | 2 | | 0.729 |
| 24 | b33 | Unknow | 15 | 0.714 | 30 | | 0.676 | 4 | | 0.758 | 23 | | 0.652 |
| 25 | b34 | 2,7-dihydroxy-1-(4-hydroxybenzyl)-4-methoxyphenanthrene | 1 | 0.818 | 15 | | 0.72 | 10 | | 0.73 | 4 | | 0.72 |
| 26 | b36 | blestriarene A | 7 | 0.75 | 5 | | 0.748 | 12 | | 0.705 | 3 | | 0.723 |
| 27 | b37 | 2-(*p*-hydroxybenzyl)-3’,5-dihydroxy-3-methoxybibenzyl | 22 | 0.677 | 7 | | 0.742 | 24 | | 0.65 | 12 | | 0.679 |
| 28 | b38 | blestriarene B | 24 | 0.666 | 33 | | 0.631 | 6 | | 0.744 | 13 | | 0.671 |
| 29 | b39 | monbarbatain A | 2 | 0.772 | 20 | | 0.702 | 3 | | 0.787 | 9 | | 0.688 |
| 30 | b40 | Unknow | 11 | 0.721 | 9 | | 0.734 | 18 | | 0.684 | 8 | | 0.694 |
| 31 | b41 | Unknow | 19 | 0.695 | 8 | | 0.736 | 16 | | 0.69 | 10 | | 0.686 |
| 32 | b42 | Unknow | 17 | 0.702 | 10 | | 0.734 | 15 | | 0.694 | 6 | | 0.7 |
| 33 | b43 | Unknow | 5 | 0.758 | 22 | | 0.698 | 8 | | 0.742 | 21 | | 0.654 |
| 34 | b45 | Unknow | 20 | 0.688 | 25 | | 0.694 | 21 | | 0.669 | 19 | | 0.661 |

**Table S7.** Correlations coefficient and rank of GRA analysis of Maocigu.

| NO. | Peak | Components | HepG2  proliferation inhibition | | | HepG2  migration inhibition | | | Huh7  proliferation inhibition | | | Huh7  migration inhibition | |
| --- | --- | --- | --- | --- | --- | --- | --- | --- | --- | --- | --- | --- | --- |
|  |  |  | Rank | Correlation coefficient | Rank | | Correlation coefficient | Rank | | Correlation coefficient | Rank | | Correlation coefficient |
| 1 | m1 | gastrodin | 7 | 0.834 | 13 | | 0.764 | 21 | | 0.788 | 4 | | 0.809 |
| 2 | m2 | 2-isobutylmalic acid | 1 | 0.883 | 17 | | 0.757 | 15 | | 0.807 | 5 | | 0.808 |
| 3 | m3 | Unknow | 5 | 0.84 | 5 | | 0.817 | 11 | | 0.82 | 18 | | 0.749 |
| 4 | m5 | loroglossin | 10 | 0.811 | 1 | | 0.849 | 12 | | 0.819 | 12 | | 0.764 |
| 5 | m6 | dactylorhin A | 30 | 0.633 | 30 | | 0.686 | 29 | | 0.724 | 24 | | 0.728 |
| 6 | m7 | gymnoside | 17 | 0.765 | 25 | | 0.73 | 1 | | 0.863 | 7 | | 0.792 |
| 7 | m8 | gymnoside II | 6 | 0.838 | 11 | | 0.765 | 3 | | 0.857 | 6 | | 0.802 |
| 8 | m10 | Unknow | 28 | 0.674 | 19 | | 0.752 | 28 | | 0.735 | 21 | | 0.738 |
| 9 | m12 | Unknow | 24 | 0.754 | 14 | | 0.762 | 27 | | 0.736 | 25 | | 0.712 |
| 10 | m13 | Unknow | 15 | 0.766 | 2 | | 0.825 | 24 | | 0.768 | 29 | | 0.689 |
| 11 | m14 | militarine | 12 | 0.802 | 4 | | 0.818 | 23 | | 0.777 | 26 | | 0.708 |
| 12 | m15 | 1,4-di(4-*β*-D-glucopyranosyloxybenzyl)-2-benzylmalate | 3 | 0.861 | 6 | | 0.811 | 10 | | 0.823 | 19 | | 0.747 |
| 13 | m16 | Unknow | 22 | 0.759 | 7 | | 0.782 | 8 | | 0.83 | 20 | | 0.743 |
| 14 | m18 | coelonin | 23 | 0.756 | 22 | | 0.746 | 2 | | 0.858 | 15 | | 0.76 |
| 15 | m23 | gymnoside IV | 20 | 0.762 | 3 | | 0.821 | 13 | | 0.808 | 1 | | 0.857 |
| 16 | m24 | gymnoside V isomer | 27 | 0.676 | 21 | | 0.748 | 30 | | 0.712 | 16 | | 0.759 |
| 17 | m27 | Unknow | 26 | 0.753 | 16 | | 0.758 | 16 | | 0.804 | 11 | | 0.77 |
| 18 | m28 | batatasin III | 19 | 0.763 | 10 | | 0.765 | 4 | | 0.854 | 9 | | 0.774 |
| 19 | m29 | Unknow | 9 | 0.813 | 26 | | 0.728 | 9 | | 0.824 | 3 | | 0.81 |
| 20 | m30 | 2,7-dihydroxy-1-(4-hydroxybenzyl)-4-methoxyphenanthrene | 18 | 0.764 | 24 | | 0.742 | 18 | | 0.797 | 8 | | 0.788 |
| 21 | m32 | Unknow | 4 | 0.843 | 9 | | 0.773 | 20 | | 0.794 | 17 | | 0.75 |
| 22 | m33 | blestriarene A | 2 | 0.862 | 15 | | 0.76 | 5 | | 0.836 | 2 | | 0.815 |
| 23 | m34 | 2-(*p*-hydroxybenzyl)-3’,5-dihydroxy-3-methoxybibenzyl | 13 | 0.793 | 8 | | 0.775 | 14 | | 0.807 | 23 | | 0.729 |
| 24 | m35 | blestriarene B | 14 | 0.784 | 23 | | 0.744 | 22 | | 0.779 | 28 | | 0.691 |
| 25 | m36 | monbarbatain A | 16 | 0.766 | 12 | | 0.764 | 7 | | 0.83 | 13 | | 0.763 |
| 26 | m37 | Unknow | 11 | 0.808 | 27 | | 0.712 | 25 | | 0.758 | 27 | | 0.698 |
| 27 | m38 | Unknow | 8 | 0.821 | 18 | | 0.754 | 19 | | 0.796 | 22 | | 0.735 |
| 28 | m39 | Unknow | 21 | 0.762 | 28 | | 0.706 | 6 | | 0.833 | 10 | | 0.773 |
| 29 | m41 | Unknow | 25 | 0.754 | 20 | | 0.749 | 17 | | 0.801 | 14 | | 0.761 |
| 30 | m42 | Unknow | 29 | 0.642 | 29 | | 0.704 | 26 | | 0.743 | 30 | | 0.664 |

**Table S8.** Correlations coefficient and rank of BCA analysis of Bingqiuzi.

| NO. | Peak | Components | HepG2  proliferation inhibition | | | HepG2  migration inhibition | | | Huh7  proliferation inhibition | | | Huh7  migration inhibition | |
| --- | --- | --- | --- | --- | --- | --- | --- | --- | --- | --- | --- | --- | --- |
|  |  |  | Rank | Correlation coefficient | Rank | | Correlation coefficient | Rank | | Correlation coefficient | Rank | | Correlation coefficient |
| 1 | b1 | gastrodin | 11 | 0.026 | 18 | | -0.087 | 21 | | -0.442 | 23 | | -0.599 |
| 2 | b2 | 2-isobutylmalic acid | 15 | -0.057 | 26 | | -0.181 | 23 | | -0.455 | 25 | | -0.625 |
| 3 | b3 | Unknow | 27 | 0.126 | 34 | | -0.374 | 3 | | 0.382 | 5 | | 0.131 |
| 4 | b4 | loroglossin | 8 | 0.327 | 28 | | -0.217 | 8 | | 0.091 | 10 | | -0.274 |
| 5 | b5 | dactylorhin A | 16 | -0.092 | 3 | | 0.286 | 16 | | -0.297 | 18 | | -0.528 |
| 6 | b6 | gymnoside | 17 | -0.103 | 4 | | 0.103 | 18 | | -0.309 | 29 | | -0.685^*^ |
| 7 | b7 | gymnoside II | 13 | -0.005 | 20 | | -0.115 | 11 | | -0.115 | 15 | | -0.483 |
| 8 | b9 | Unknow | 29 | -0.246 | 16 | | -0.077 | 31 | | -0.624 | 30 | | -0.690^*^ |
| 9 | b10 | militarine | 27 | -0.229 | 11 | | -0.044 | 28 | | -0.588 | 32 | | -0.782^**^ |
| 10 | b12 | Unknow | 32 | -0.487 | 31 | | -0.276 | 32 | | -0.685^*^ | 33 | | -0.783^**^ |
| 11 | b13 | shancigusin H | 25 | -0.223 | 13 | | -0.045 | 22 | | -0.442 | 17 | | -0.526 |
| 12 | b14 | Unknow | 33 | -0.515 | 21 | | -0.139 | 34 | | -0.855^**^ | 34 | | -0.830^**^ |
| 13 | b15 | coelonin | 13 | 0.146 | 33 | | -0.365 | 9 | | -0.006 | 8 | | -0.194 |
| 14 | b16 | Unknow | 23 | -0.196 | 10 | | -0.020 | 27 | | -0.552 | 9 | | -0.207 |
| 15 | b19 | Unknow | 18 | -0.115 | 32 | | -0.297 | 4 | | 0.358 | 2 | | 0.297 |
| 16 | b22 | Unknow | 24 | -0.219 | 6 | | 0.012 | 33 | | -0.758^*^ | 28 | | -0.676^*^ |
| 17 | b23 | gymnoside V | 28 | -0.240 | 15 | | -0.057 | 29 | | -0.612 | 20 | | -0.560 |
| 18 | b24 | Unknow | 12 | 0.011 | 2 | | 0.316 | 17 | | -0.297 | 13 | | -0.380 |
| 19 | b25 | gymnoside VI | 10 | 0.059 | 1 | | 0.362 | 19 | | -0.345 | 12 | | -0.332 |
| 20 | b26 | gymnoside IV | 22 | -0.184 | 7 | | -0.003 | 30 | | -0.612 | 19 | | -0.541 |
| 21 | b27 | gymnoside V isomer | 6 | 0.099 | 8 | | -0.014 | 2 | | 0.612 | 1 | | 0.350 |
| 22 | b31 | batatasin III | 34 | -0.552 | 22 | | -0.139 | 24 | | -0.527 | 31 | | -0.745^*^ |
| 23 | b32 | Unknow | 18 | 0.082 | 5 | | 0.038 | 1 | | 0.673^*^ | 3 | | 0.223 |
| 24 | b33 | Unknow | 15 | 0.283 | 27 | | -0.187 | 7 | | 0.176 | 7 | | -0.114 |
| 25 | b34 | 2,7-dihydroxy-1-(4-hydroxybenzyl)-4-methoxyphenanthrene | 1 | 0.278 | 24 | | -0.166 | 15 | | -0.285 | 14 | | -0.456 |
| 26 | b36 | blestriarene A | 26 | -0.227 | 30 | | -0.264 | 13 | | -0.248 | 24 | | -0.612 |
| 27 | b37 | 2-(*p*-hydroxybenzyl)-3’,5-dihydroxy-3-methoxybibenzyl | 31 | -0.339 | 23 | | -0.149 | 25 | | -0.527 | 27 | | -0.654^*^ |
| 28 | b38 | blestriarene B | 14 | -0.030 | 9 | | -0.018 | 6 | | 0.309 | 4 | | 0.176 |
| 29 | b39 | monbarbatain A | 2 | 0.212 | 17 | | -0.079 | 5 | | 0.333 | 6 | | 0.018 |
| 30 | b40 | Unknow | 21 | -0.174 | 25 | | -0.175 | 20 | | -0.406 | 26 | | -0.627 |
| 31 | b41 | Unknow | 19 | -0.133 | 14 | | -0.045 | 12 | | -0.236 | 22 | | -0.588 |
| 32 | b42 | Unknow | 20 | -0.173 | 19 | | -0.112 | 14 | | -0.261 | 16 | | 0.7 |
| 33 | b43 | Unknow | 5 | 0.205 | 12 | | -0.044 | 10 | | -0.006 | 11 | | 0.654 |
| 34 | b45 | Unknow | 30 | -0.300 | 29 | | -0.221 | 26 | | -0.539 | 21 | | 0.661 |

Note: ^*^*P* < 0.05, ^**^*P* < 0.01.

**Table S9.** Correlations coefficient and rank of BCA analysis of Maocigu.

| NO. | Peak | Components | HepG2  proliferation inhibition | | | HepG2  migration inhibition | | | Huh7  proliferation inhibition | | | Huh7  migration inhibition | |
| --- | --- | --- | --- | --- | --- | --- | --- | --- | --- | --- | --- | --- | --- |
|  |  |  | Rank | Correlation coefficient | Rank | | Correlation coefficient | Rank | | Correlation coefficient | Rank | | Correlation coefficient |
| 1 | m1 | gastrodin | 4 | 0.680^*^ | 11 | | 0.164 | 20 | | 0.095 | 11 | | 0.164 |
| 2 | m2 | 2-isobutylmalic acid | 1 | 0.808^**^ | 10 | | 0.188 | 18 | | 0.176 | 10 | | 0.188 |
| 3 | m3 | Unknow | 9 | 0.522 | 1 | | 0.612 | 16 | | 0.206 | 1 | | 0.612 |
| 4 | m5 | loroglossin | 18 | 0.219 | 2 | | 0.564 | 21 | | 0.079 | 2 | | 0.564 |
| 5 | m6 | dactylorhin A | 30 | -0.636^*^ | 25 | | -0.139 | 25 | | -0.067 | 25 | | -0.139 |
| 6 | m7 | gymnoside | 23 | -0.028 | 28 | | -0.297 | 11 | | 0.372 | 28 | | -0.297 |
| 7 | m8 | gymnoside II | 10 | 0.471 | 15 | | 0.103 | 4 | | 0.487 | 15 | | 0.103 |
| 8 | m10 | Unknow | 27 | -0.345 | 16 | | 0.079 | 27 | | -0.127 | 16 | | 0.079 |
| 9 | m12 | Unknow | 21 | 0.063 | 20 | | 0.006 | 29 | | -0.408 | 20 | | 0.006 |
| 10 | m13 | Unknow | 14 | 0.289 | 3 | | 0.442 | 23 | | -0.017 | 3 | | 0.442 |
| 11 | m14 | militarine | 16 | 0.279 | 5 | | 0.418 | 24 | | -0.021 | 5 | | 0.418 |
| 12 | m15 | 1,4-di(4-*β*-D-glucopyranosyloxybenzyl)-2-benzylmalate | 5 | 0.663^*^ | 6 | | 0.418 | 13 | | 0.298 | 6 | | 0.418 |
| 13 | m16 | Unknow | 19 | 0.106 | 8 | | 0.285 | 6 | | 0.420 | 8 | | 0.285 |
| 14 | m18 | coelonin | 26 | -0.280 | 22 | | -0.030 | 9 | | 0.373 | 22 | | -0.030 |
| 15 | m23 | gymnoside IV | 25 | -0.255 | 7 | | 0.309 | 26 | | -0.116 | 7 | | 0.309 |
| 16 | m24 | gymnoside V isomer | 28 | -0.451 | 24 | | -0.115 | 30 | | -0.606 | 24 | | -0.115 |
| 17 | m27 | Unknow | 17 | 0.227 | 26 | | -0.152 | 19 | | 0.124 | 26 | | -0.152 |
| 18 | m28 | batatasin III | 24 | -0.051 | 18 | | 0.067 | 10 | | 0.373 | 18 | | 0.067 |
| 19 | m29 | Unknow | 13 | 0.371 | 29 | | -0.382 | 17 | | 0.200 | 29 | | -0.382 |
| 20 | m30 | 2,7-dihydroxy-1-(4-hydroxybenzyl)-4-methoxyphenanthrene | 15 | 0.285 | 27 | | -0.164 | 14 | | 0.285 | 27 | | -0.164 |
| 21 | m32 | Unknow | 8 | 0.589 | 17 | | 0.079 | 22 | | 0.072 | 17 | | 0.079 |
| 22 | m33 | blestriarene A | 3 | 0.700^*^ | 14 | | 0.115 | 3 | | 0.501 | 14 | | 0.115 |
| 23 | m34 | 2-(*p*-hydroxybenzyl)-3’,5-dihydroxy-3-methoxybibenzyl | 7 | 0.641^*^ | 4 | | 0.430 | 8 | | 0.387 | 4 | | 0.430 |
| 24 | m35 | blestriarene B | 12 | 0.442 | 13 | | 0.139 | 5 | | 0.430 | 13 | | 0.139 |
| 25 | m36 | monbarbatain A | 11 | 0.455 | 9 | | 0.212 | 1 | | 0.685^*^ | 9 | | 0.212 |
| 26 | m37 | Unknow | 2 | 0.758^*^ | 19 | | 0.030 | 2 | | 0.503 | 19 | | 0.030 |
| 27 | m38 | Unknow | 6 | 0.650^*^ | 21 | | -0.006 | 12 | | 0.307 | 21 | | -0.006 |
| 28 | m39 | Unknow | 20 | 0.102 | 23 | | -0.055 | 7 | | 0.410 | 23 | | -0.055 |
| 29 | m41 | Unknow | 22 | 0.015 | 12 | | 0.164 | 15 | | 0.233 | 12 | | 0.164 |
| 30 | m42 | Unknow | 29 | -0.624 | 30 | | -0.552 | 28 | | -0.164 | 30 | | -0.552 |

Note: ^*^*P* < 0.05, ^**^*P* < 0.01.

**Table S10.** The content and proportion of each active component in Bingqiuzi

| **Active component** | **Content of crude BQZ-3 (μg·g^-1^)** | **Proportion (%)** |
| --- | --- | --- |
| monbarbatain A | 4.19 | 0.008 |
| blestriarene A | 35.06 | 0.066 |
| blestriarene B | 20.56 | 0.039 |
| 2,7-dihydroxy-1-(4-hydroxybenzyl)-4-methoxyphenanthrene | 4.56 | 0.009 |
| coelonin | 128.8 | 0.244 |
| batatasin III | 197.03 | 0.373 |
| 2-(*p*-hydroxybenzyl)-3’,5-dihydroxy-3-methoxybibenzyl | 294.81 | 0.558 |
| gastrodin | 22713.24 | 42.997 |
| 2-isobutylmalic acid | 5569.23 | 10.543 |
| malic acid | 20676.03 | 39.14 |
| citric acid | 3181.66 | 6.023 |

**Table S11.** The content and proportion of each active component in Maocigu

| **Active component** | **Content of crude MCG-9 (μg·g^-1^)** | **Proportion (%)** |
| --- | --- | --- |
| monbarbatain A | 12.47 | 0.039 |
| blestriarene A | 24.47 | 0.077 |
| blestriarene B | 33.91 | 0.107 |
| 2,7-dihydroxy-1-(4-hydroxybenzyl)-4-methoxyphenanthrene | 7.11 | 0.022 |
| coelonin | 100.72 | 0.317 |
| batatasin III | 111.94 | 0.353 |
| 2-(*p*-hydroxybenzyl)-3’,5-dihydroxy-3-methoxybibenzyl | 308.96 | 0.974 |
| gastrodin | 14119.43 | 44.504 |
| 2-isobutylmalic acid | 4447.74 | 14.019 |
| malic acid | 11567.88 | 36.461 |
| citric acid | 991.65 | 3.126 |

**Table S12.** Comparison of tumor inhibition rates among different groups.

| **Groups** | **Inbition rates of liver cancer（%）** |
| --- | --- |
| Y | 65.04 |
| MCG-9 | 60.93 |
| MCGC | 60.99 |
| BQZ-3 | 62.48 |
| BQZC | 62.32 |

**Table S13.** Content determination of 11 effective components in 26 batches of Shancigu.

| **NO.** | **Mass fraction（µg·g^-1^）** | | | | | | | | | | |
| --- | --- | --- | --- | --- | --- | --- | --- | --- | --- | --- | --- |
|  | **A** | **B** | **C** | **D** | **E** | **F** | **G** | **H** | **I** | **J** | **K** |
| BQZ-1 | 8900.15 | 696.10 | 17309.39 | 5038.17 | 151.24 | 731.50 | 237.78 | 21.87 | 85.29 | 70.68 | 19.31 |
| BQZ-2 | 7025.15 | 784.81 | 5966.87 | 2809.95 | 216.07 | 728.45 | 306.18 | 19.74 | 86.50 | 58.24 | 16.54 |
| BQZ-3 | 8818.43 | 820.42 | 7541.36 | 2854.53 | 282.06 | 913.74 | 397.88 | 29.52 | 106.52 | 100.29 | 28.42 |
| BQZ-4 | 9374.67 | 1562.29 | 10580.73 | 3202.56 | 172.13 | 250.85 | 218.11 | 8.08 | 27.38 | 34.29 | 8.96 |
| BQZ-5 | 7992.39 | 1732.55 | 7464.69 | 1754.95 | 257.54 | 295.25 | 417.05 | 6.84 | 29.60 | 40.34 | 9.06 |
| BQZ-6 | 8539.57 | 2199.79 | 9194.00 | 2139.10 | 269.17 | 327.56 | 386.66 | 8.54 | 36.08 | 45.15 | 11.50 |
| BQZ-7 | 7103.96 | 745.55 | 11477.58 | 3661.67 | 56.89 | 219.96 | 62.86 | 5.61 | 16.30 | 23.10 | 8.22 |
| BQZ-8 | 11163.17 | 1046.21 | 11555.69 | 3376.62 | 127.74 | 217.28 | 102.46 | 5.08 | 14.32 | 23.58 | 9.21 |
| BQZ-9 | 11567.88 | 991.65 | 14119.43 | 4447.74 | 111.94 | 308.96 | 100.72 | 7.11 | 24.47 | 33.91 | 12.47 |
| BQZ-10 | 10985.52 | 1271.04 | 13416.59 | 3720.75 | 277.76 | 372.90 | 247.13 | 7.93 | 29.04 | 33.75 | 10.89 |
| BQZ-11 | 10138.63 | 1342.07 | 9227.57 | 2685.16 | 220.11 | 323.11 | 242.42 | 8.67 | 21.39 | 25.94 | 8.04 |
| BQZ-12 | 11083.14 | 1518.95 | 10926.74 | 3649.56 | 293.91 | 411.21 | 293.04 | 7.00 | 37.82 | 34.49 | 10.77 |
| BQZ-13 | 16473.96 | 2040.04 | 17732.41 | 4521.18 | 118.52 | 214.85 | 91.22 | 4.31 | 25.03 | 13.46 | 2.80 |
| BQZ-14 | 19785.20 | 2893.64 | 18433.02 | 5113.68 | 207.00 | 81.62 | 29.99 | 0.46 | 4.12 | 2.41 | 0.64 |
| MCG-1 | 20676.03 | 3181.66 | 22713.24 | 5569.23 | 197.03 | 294.81 | 128.80 | 4.56 | 35.06 | 20.56 | 4.19 |
| MCG-2 | 33736.73 | 3735.87 | 20519.71 | 5325.95 | 185.02 | 98.37 | 36.75 | 5.70 | 4.58 | 4.35 | 0.78 |
| MCG-3 | 16904.76 | 2262.74 | 15610.38 | 3485.99 | 67.27 | 74.12 | 21.37 | 1.71 | 4.55 | 2.53 | 0.63 |
| MCG-4 | 25248.59 | 2019.25 | 19230.86 | 4511.53 | 79.94 | 79.36 | 22.77 | 1.77 | 3.42 | 2.28 | 0.57 |
| MCG-5 | 15826.29 | 3392.97 | 17414.03 | 3733.38 | 118.28 | 107.48 | 46.98 | 2.72 | 6.76 | 3.69 | 1.12 |
| MCG-6 | 37734.49 | 4191.91 | 17414.03 | 3727.38 | 528.04 | 147.28 | 48.96 | 1.31 | 4.98 | 3.30 | 0.77 |
| MCG-7 | 39784.08 | 3152.86 | 29239.91 | 7191.88 | 361.73 | 133.19 | 39.19 | 1.90 | 4.80 | 4.34 | 0.88 |
| MCG-8 | 50148.78 | 6601.81 | 26417.33 | 6983.07 | 560.28 | 168.01 | 63.21 | 2.35 | 5.86 | 4.54 | 1.10 |
| MCG-9 | 44749.79 | 2673.31 | 23479.71 | 5318.35 | 234.08 | 203.62 | 86.81 | 3.71 | 9.18 | 6.99 | 1.43 |
| MCG-10 | 45262.98 | 3139.51 | 23853.09 | 5549.82 | 266.83 | 206.28 | 76.47 | 3.12 | 8.62 | 7.16 | 1.30 |
| MCG-11 | 47746.65 | 3681.02 | 24906.59 | 6676.19 | 265.19 | 150.92 | 38.35 | 2.77 | 4.10 | 4.41 | 0.78 |
| MCG-12 | 48491.75 | 4052.62 | 28771.78 | 6381.30 | 384.90 | 243.09 | 122.80 | 4.43 | 11.31 | 6.92 | 1.60 |

Note: A-malic acid B-citric acid C-2-isobutylmalic acid D-gastrodin

E-batatasin III F-2-(*p*-hydroxybenzyl)-3’,5-dihydroxy-3-methoxybibenzyl

G-coelonin H-2,7-dihydroxy-1-(4-hydroxybenzyl)-4-methoxyphenanthrene

I-blestriarene A J-blestriarene B K-monbarbatain A

**Table S14.** Comprehensive pharmacodynamic indexes of 20 batches of different commercial specifications of Shancigu against liver cancer based on entropy weight method.

| **Sample** | **Comprehensive pharmacodynamic indexes** |
| --- | --- |
| BQZ-1 | 0.370 |
| BQZ-2 | 0.263 |
| BQZ-3 | 0.804 |
| BQZ-4 | 0.241 |
| BQZ-5 | 0.937 |
| BQZ-6 | 0.499 |
| BQZ-7 | 0.622 |
| BQZ-8 | 0.322 |
| BQZ-9 | 0.450 |
| BQZ-10 | 0.404 |
| MCG-1 | 0.500 |
| MCG-4 | 0.205 |
| MCG-5 | 0.202 |
| MCG-6 | 0.262 |
| MCG-7 | 0.295 |
| MCG-8 | 0.253 |
| MCG-9 | 0.679 |
| MCG-10 | 0.431 |
| MCG-11 | 0.371 |
| MCG-12 | 0.389 |

**Table S15.** Contribution and ranking of 11 effective components of Shancigu against liver cancer based on GRA.

| **Components** | **Contribution against liver cancer efficacy** | **Rank** |
| --- | --- | --- |
| malic acid | 0.624 | 10 |
| citric acid | 0.68 | 6 |
| 2-isobutylmalic acid | 0.742 | 1 |
| gastrodin | 0.717 | 2 |
| batatasin III | 0.686 | 5 |
| 2-(p-hydroxybenzyl)-3’,5-  dihydroxy-3-methoxybibenzyl | 0.697 | 4 |
| coelonin | 0.612 | 11 |
| 2,7-dihydroxy-1-(4-hydroxybenzyl)-4-methoxyphenanthrene | 0.697 | 3 |
| blestriarene A | 0.661 | 7 |
| blestriarene B | 0.655 | 8 |
| monbarbatain A | 0.637 | 9 |

**Table S16.** Results of TOPSIS analysis of 26 batches Shancigu samples.

| **NO.** | **D^+^** | **D^-^** | **Comprehensive quality score** |
| --- | --- | --- | --- |
| BQZ-1 | 0.78434 | 0.270823 | 0.256664 |
| BQZ-2 | 0.82845 | 0.307189 | 0.270499 |
| BQZ-3 | 0.682446 | 0.398351 | 0.368572 |
| BQZ-4 | 0.771706 | 0.383429 | 0.331934 |
| BQZ-5 | 0.889857 | 0.194621 | 0.179461 |
| BQZ-6 | 0.856896 | 0.277897 | 0.244888 |
| BQZ-7 | 0.841697 | 0.24883 | 0.228174 |
| BQZ-8 | 0.76464 | 0.44026 | 0.365391 |
| BQZ-9 | 0.740174 | 0.544365 | 0.423782 |
| BQZ-10 | 0.690098 | 0.662275 | 0.489713 |
| BQZ-11 | 0.733516 | 0.436235 | 0.37293 |
| BQZ-12 | 0.725191 | 0.457425 | 0.386791 |
| BQZ-13 | 0.741054 | 0.51202 | 0.408611 |
| BQZ-14 | 0.665312 | 0.569337 | 0.461133 |
| MCG-1 | 0.565799 | 0.578466 | 0.505535 |
| MCG-2 | 0.659888 | 0.519075 | 0.440281 |
| MCG-3 | 0.584553 | 0.741528 | 0.559188 |
| MCG-4 | 0.760743 | 0.266651 | 0.259541 |
| MCG-5 | 0.770674 | 0.372471 | 0.32583 |
| MCG-6 | 0.717965 | 0.387619 | 0.350601 |
| MCG-7 | 0.853173 | 0.190602 | 0.182609 |
| MCG-8 | 0.820726 | 0.198636 | 0.194863 |
| MCG-9 | 0.756911 | 0.288019 | 0.275635 |
| MCG-10 | 0.700296 | 0.337456 | 0.32518 |
| MCG-11 | 0.772527 | 0.267836 | 0.257445 |
| MCG-12 | 0.69126 | 0.359864 | 0.342361 |

**Table S17.** Results of TOPSIS analysis of 14 batches Bingqiuzi samples from different areas.

| **NO.** | **D^+^** | **D^-^** | **Comprehensive quality score** |
| --- | --- | --- | --- |
| BQZ-1 | 0.63681997 | 0.50889749 | 0.44417364 |
| BQZ-2 | 0.88784308 | 0.17968232 | 0.16831667 |
| BQZ-3 | 0.43892591 | 0.77469894 | 0.63833477 |
| BQZ-4 | 0.73962036 | 0.42309547 | 0.36388553 |
| BQZ-5 | 0.96458133 | 0.07956014 | 0.0761967 |
| BQZ-6 | 0.9075132 | 0.15936915 | 0.14937837 |
| BQZ-7 | 0.84150659 | 0.20385768 | 0.19501114 |
| BQZ-8 | 0.77072146 | 0.38225515 | 0.33153765 |
| BQZ-9 | 0.6910541 | 0.51855857 | 0.42869803 |
| BQZ-10 | 0.57367822 | 0.65710144 | 0.5338904 |
| BQZ-11 | 0.59206449 | 0.49305084 | 0.45437644 |
| BQZ-12 | 0.58847571 | 0.48662552 | 0.45263228 |
| BQZ-13 | 0.67810325 | 0.48366767 | 0.41631931 |
| BQZ-14 | 0.44891762 | 0.69641866 | 0.60804732 |

**Table S18.** Results of TOPSIS analysis of 12 batches Maocigu samples from different areas.

| **NO.** | **D^+^** | **D^-^** | **Comprehensive quality score** |
| --- | --- | --- | --- |
| MCG-1 | 0.47530264 | 0.67069023 | 0.58524817 |
| MCG-2 | 0.6431711 | 0.51220822 | 0.44332472 |
| MCG-3 | 0.47247739 | 0.79722101 | 0.62788219 |
| MCG-4 | 0.72174469 | 0.36176126 | 0.33388027 |
| MCG-5 | 0.76633309 | 0.45137691 | 0.37067685 |
| MCG-6 | 0.67889133 | 0.51664857 | 0.43214666 |
| MCG-7 | 0.91270763 | 0.23308236 | 0.20342503 |
| MCG-8 | 0.81014768 | 0.37299486 | 0.31525775 |
| MCG-9 | 0.72620533 | 0.47603887 | 0.39595855 |
| MCG-10 | 0.63904872 | 0.51539257 | 0.44644329 |
| MCG-11 | 0.74168256 | 0.37740245 | 0.33724199 |
| MCG-12 | 0.62143397 | 0.53626578 | 0.46321663 |
